# TCF12 promotes the tumorigenesis and metastasis of hepatocellular carcinoma via upregulation of CXCR4 expression

**DOI:** 10.7150/thno.34973

**Published:** 2019-08-12

**Authors:** Jing Yang, Lili Zhang, Zhiyuan Jiang, Chao Ge, Fangyu Zhao, Jingyi Jiang, Hua Tian, Taoyang Chen, Haiyang Xie, Ying Cui, Ming Yao, Hong Li, Jinjun Li

**Affiliations:** 1 State Key Laboratory of Oncogenes and Related Genes, Shanghai Cancer Institute, Renji Hospital, Shanghai Jiaotong University School of Medicine, Shanghai 200032, China; 2 Testing Center, Center for Disease Prevention and Control, Changzhou 213000, China; 3 Qi Dong Liver Cancer Institute, Qi Dong 226200, China; 4 Department of General Surgery, the First Affiliated Hospital, School of Medicine, Zhejiang University, Hangzhou 310000, China; 5 Cancer Institute of Guangxi, Nanning 530027, China

**Keywords:** hepatocellular carcinoma, TCF12, CXCR4, CXCL12

## Abstract

TCF12, which is known to be involved in the regulation of cell growth and differentiation, has been reported to function as an oncogene or a tumor suppressor gene in the progression of various malignant tumors. However, its function and molecular mechanism in hepatocellular carcinoma (HCC) remain unclear.

**Methods:** Stable ectopic TCF12 expression or knockdown in HCC cell lines was established by lentiviral infection. Then, MTT, colony formation, migration, invasion and HUVECs tube formation assays as well as an orthotopic xenograft model were used to investigate the biologic function of TCF12 in HCC cells *in vitro* and* in vivo*. Subsequently, RNA-Seq analysis was utilized to explore the target genes regulated by TCF12. RT-qPCR, western blotting, a dual-luciferase reporter assay, Ch-IP, CHIP-Seq and functional rescue experiments were used to confirm the target gene regulated by TCF12. Finally, RT-qPCR, western blot and immunohistochemical (IHC) staining were performed to detect the expression level of TCF12 and to analyze the correlation of TCF12 with downstream genes as well as the clinical significance of TCF12 in human primary HCC.

**Results:** Our functional studies revealed that stable overexpression of TCF12 in human HCC cells enhanced cell proliferation, migration and invasion *in vitro* and *in vivo*, whereas knockdown of TCF12 showed opposing effects. Mechanistically, CXCR4 was a downstream target of TCF12, and TCF12 directly bound to the CXCR4 promoter to regulate its expression. Moreover, CXCR4, with its ligand CXCL12, played a critical role in tumor progression induced by TCF12 via activation of the MAPK/ERK and PI3K/AKT signaling pathways. Clinically, IHC analysis revealed that TCF12 was significantly associated with poor survival of HCC patients and that TCF12 expression was closely correlated with CXCR4 expression in primary HCC tissues.

**Conclusion:** Our findings are the first to indicate that TCF12 could promote the tumorigenesis and progression of HCC mainly by upregulating CXCR4 expression and is a prognostic indicator for patients with HCC.

## Introduction

Liver cancer is one of the most common malignancies, ranking sixth globally in morbidity and fourth in mortality, with approximately 841,000 new cases and 782,000 patient deaths each year 1. Hepatocellular carcinoma (HCC) is the most common type of primary liver cancer, and the major risk factors for HCC are hepatitis B virus or hepatitis C virus infection, aflatoxin exposure and heavy alcohol consumption 2. Despite improvements in treatments for this disease, the prognosis and survival rate for primary HCC patients remain dismal due to diagnosis at advanced stage and limited effective therapeutic options 3, 4. Therefore, it is essential to more thoroughly understand the molecular mechanisms underlying HCC and to find more effective therapeutic strategies.

The basic helix-loop-helix (bHLH) family of transcription factors, all of which contain two distinct motifs, the HLH domain and the adjacent basic region, are involved in cell growth and differentiation processes, including myogenesis, neurogenesis and lymphocyte differentiation 5, 6. The bHLH protein family can be divided into the non-tissue-specific expressed class A proteins and tissue-specific expressed class B proteins 7. The class A proteins, also called E proteins for their direct DNA (E-box, CANNTG) binding ability, consist of TCF3, TCF4 and TCF12. TCF12 can form homodimers or heterodimers with other family members to regulate cell development and differentiation in multiple tissues, such as skeletal muscle, neurons, mesenchymal tissues and lymphocytes 9-13. Recently, TCF12 has been show to function as an oncogene or a tumor suppressor gene in tumor progression. In colorectal cancer (CRC), TCF12 was significantly correlated with the occurrence of metastasis through repression of the E-cadherin gene promoter 14, 15. In addition, TCF12 can increase the invasion of cells and positively correlates with distant metastasis and poor overall survival (OS) of gallbladder cancer (GBC) 16. In breast cancer, TCF12 mediated the activation of cancer-associated fibroblasts (CAFs) and contributed to extracellular matrix (ECM) remodeling, triggering the invasion and metastasis of breast cancer cells both *in vitro* and *in vivo* 17. In contrast, other studies revealed that TCF12 mediated suppressor activities and was drastically downregulated in oral squamous cell carcinoma and prostate cancer 18, 19. Nevertheless, the function and molecular mechanism of TCF12 in HCC have never been reported until now.

Our work identified C‑X‑C chemokine receptor 4 (CXCR4) as a direct target of TCF12. Numerous studies have demonstrated that CXCR4 and its ligand, chemokine ligand 12 (CXCL12), are implicated in processes relating to the progression of HCC, including cell proliferation, angiogenesis, invasion and metastasis 20-23. For example, elevated EphA1 expression in HCC cells enhanced the recruitment of endothelial progenitor cells (EPCs) to the tumor site to promote angiogenesis via activation of the CXCL12/ CXCR4 axis 24. High levels of CXCR4 in tumor endothelial cells could induce tumor angiogenesis, which facilitates tumor metastasis and correlates with poor prognosis of HCC patients 25. Furthermore, increased cytoplasmic expression of CXCR4 showed significantly worse OS, and aberrant CXCR4 overexpression in HCC cells could promote cell proliferation and migration 22. In addition, studies have also shown that CXCR4 expression could be induced in activated hepatic stellate cells (HSCs) during the progression of liver fibrosis, and CXCR4-targeted nanoparticles combined with sorafenib and MEK inhibitors could reduce liver fibrosis and prevent HCC development 26, 27. Despite these studies, the relationship between CXCR4 expression and TCF12 in HCC has long been unclear.

In the present study, our functional experiments revealed that TCF12 could enhance HCC cell proliferation and migration *in vitro* and tumor formation *in vivo*. The protumorigenic functions induced by TCF12 were exerted via upregulation of CXCR4, thus leading to the activation of the MAPK/ERK and PI3K/AKT signaling pathways in HCC cells. Moreover, we further disclosed that CXCL12/CXCR4 mediated the effects of TCF12 on angiogenesis, and the chemotaxis of HCC cells toward CXCL12. Finally, we also observed that TCF12 was overexpressed in HCC tissues and was significantly correlated with CXCR4 and CXCL12 expression, and with worse OS. Our data suggested that targeting TCF12 might provide a therapeutic antitumor approach to HCC.

## Materials and Methods

### Cell lines and cell culture

The human HCC cell lines MHCC-97L, MHCC-97H and MHCC-LM3 were kindly donated by the Liver Cancer Institute of Zhongshan Hospital at Fudan University (Shanghai, China). The Li7 cell line was purchased from Shanghai Bioleaf Biotech Co., Ltd. SMMC-7721 cells and the immortalized human normal hepatocyte cell line L02 and MIHA were obtained from the Cell Bank of the Institute of Biochemistry and Cell Biology, Chinese Academy of Sciences (Shanghai, China). Hep3B, PLC/PRF/5, SK-Hep1, HUVEC, and HEK-293T cells were purchased from the American Type Culture Collection (ATCC) (Manassas, VA, USA). Huh7 cells were obtained from the Riken Cell Bank. All HCC cell lines were cultured in Dulbecco's modified Eagle's medium (DMEM, Gibco, New York, USA) supplemented with 10% fetal bovine serum (FBS) (Gibco, New York, USA), 100 U/mL penicillin and 100 μg/mL streptomycin (Sigma-Aldrich, St. Louis, USA) at 37 °C in a humidified atmosphere containing 5% CO_2_. All of the cell lines used in this study were regularly tested for the absence of Mycoplasma contamination and authenticated by morphological observation.

### Human HCC tissues

Paired (n=191) human primary HCC and adjacent noncancerous liver tissues were obtained from the Qidong Liver Cancer Institute and the First affiliated Hospital of Zhejiang University after approval by the University Ethical Committee. Informed consent was obtained from all patients who did not undergo any anticancer therapy prior to surgical resection. Data including 373 liver cancer patients from The Cancer Genome Atlas (TCGA) were downloaded from the website (http://cancergenome.nih.gov). OS was defined as the interval between the date of surgery and the date of either death or the last follow-up.

### Plasmid construction and transfection

The coding sequences (CDSs) of the human TCF12 and CXCR4 genes were amplified from Hep3B cells and cloned into the lentiviral vector pWPXL (Addgene, Cambridge, USA). Specific RNA interference sequences against human TCF12 were synthesized by Sangon Biotech (Shanghai, China) and cloned into the lentiviral vector pLVTHM (Addgene, Cambridge, USA). The negative control (shNC) and the short hairpin RNAs (shRNAs) targeting CXCR4 were purchased from GeneChem (Shanghai, China). The CXCR4 promoter sequence spanning 1067 bp upstream of the transcriptional start site (TSS) was amplified and cloned into the pGL3 vector (Promega, Madison, WI). The truncated (712 bp upstream of the TSS) and mutated sequences of the CXCR4 promoter were amplified from the wild-type plasmid. Primers used for PCR and target sequences are provided in Table S1. All the DNA constructs were further verified by sequencing. HEK-293T cells were transfected with a mixture of psPAX2 and pMD2.G plasmids (Addgene, Cambridge, USA) and recombinant overexpressing or interfering plasmid for lentivirus packaging using Lipofectamine™ 2000 (Invitrogen, California, USA). The lentivirus was harvested 48 h later and added to HCC cells with 6 μg/mL polybrene (Sigma-Aldrich, St. Louis, USA).

### Quantitative real-time polymerase chain reaction (RT-qPCR)

Total RNA was extracted from human primary HCC tissue specimens and cell lines using TRIzol reagent (Invitrogen, Carlsbad, CA, USA) and was reverse-transcribed with a PrimeScriptTM RT Reagent kit (TaKaRa, Dalian, China). RT-qPCR was performed with a 7500 Real-Time PCR system (Thermo Scientific, MA, USA) using SYBR Green Master Mix as described by the manufacturer's protocol (TaKaRa, Dalian, China). The mRNA expression levels were normalized to those of *GAPDH* and quantified by the comparative CT (2^-ΔΔCT^) method. All primers used for RT-qPCR are listed in Table S1.

### Western blot and enzyme-linked immunosorbent assay (ELISA)

Human primary HCC tissues and cells were lysed with RIPA buffer (Thermo Scientific, MA, USA) supplemented with a protease inhibitor cocktail and phosphatase inhibitor (Roche, Welwyn Garden, Swiss). After the proteins were quantified and denatured, samples were separated by SDS-PAGE electrophoresis and then transferred to polyvinylidene difluoride (PVDF) membranes (Millipore, Massachusetts, USA). The membranes were blocked with 5% nonfat milk solution for 1 h at room temperature, incubated with primary antibody overnight at 4 °C and then reacted with HRP-conjugated secondary antibody for 1.5 h at room temperature. The protein bands on the membranes were visualized by a Pierce ECL development system (Thermo Scientific, MA, USA) via a chemiluminescence analyzer (Bio-Rad, CA, USA). β-actin was used as an internal loading control for all the western blot experiments. The antibodies used are listed in Table S2. The CXCL12 levels in the cultured supernatants of HCC cells were measured by ELISA (R&D Systems, Minneapolis, USA) according to the manufacturer's instructions.

### *In vitro* cell proliferation and colony formation assays

Cell proliferation was measured by the MTT assay and colony formation assay. Briefly, 10^3^ cells/well were seeded in 96-well plates and incubated for the indicated times. Then, 10 μL of MTT solution (5 mg/mL, Sigma-Aldrich, St. Louis, USA) was added to each well and incubated in a CO_2_ incubator at 37 °C. After 4 h, the formazan crystals were dissolved in 100 μL of DMSO, and the absorbance at A570 was measured in a microreader (Thermo Scientific, MA, USA). For the colony formation assay, 500~1000 cells were seeded in 6-well plates for approximately 14 days. The CXCR4 antagonist AMD3100 (100 nM, Sigma-Aldrich, St. Louis, USA) was added to the wells every 48 h. Then, the colonies were fixed with 10% PBS-buffered formaldehyde and stained with crystal violet to visualize the colonies.

### *In vitro* cell migration, invasion and chemotaxis assays

HCC cells were seeded into 6-well cell culture plates at a concentration of 1 × 10^6^ cells/well and incubated for 24 h. Then, the confluent monolayer of cells was scratched with a 200-µl pipette tip and then washed twice with 1× PBS. Next, 2 mL DMEM containing 2% FBS and 1 mM thymidine (Sigma-Aldrich, St. Louis, USA) was added to each well, and the width of the scratches was measured and imaged at 0 h and 48 h. The cell invasion assays were performed in Matrigel-coated 8-µm pore membranes in 24-well transwell chambers (BD Biosciences, New Jersey, USA). A total of 1 × 10^5^ cells were suspended in serum-free medium, seeded into the upper chamber and allowed to migrate toward DMEM containing 10% FBS in the lower side of the chamber for 24~48 h. The migrated cells were fixed in formaldehyde and stained with crystal violet solution for 10 min. The cells that migrated to the lower surface of the insert membrane were counted as invaded cells under a microscope. The chemotaxis assay was similar to the invasion assay, except that the complete medium in the lower compartment was replaced with DMEM containing recombinant human CXCL12 protein ( R&D Systems, USA) as a chemoattractant.

### *In vitro* HUVEC tube formation assay

For the tube formation assay, 300 μL of growth factor-reduced Matrigel (BD Biosciences, New Jersey, USA) was added into wells of precooled 48-well plates and incubated for 30-60 min at 37 °C to allow the gel to solidify. Subsequently, HUVECs (5 x 10^4^ cells/well) were suspended in HCC cell-derived conditioned medium (CM) supplemented with 10% FBS, seeded into a 48-well plate (300 µL/well) and incubated at 37 °C for 8 h. The cells were then monitored for tube formation under an inverted light microscope.

### Mouse liver orthotopic transplantation assay

Four- to six-week-old male BALB/c nude mice were provided and housed in the Laboratory of Experimental Pathology, Shanghai Cancer Institute. All animal experimental protocols were performed in accordance with the guidelines of the Shanghai Medical Experimental Animal Care Commission. Briefly, 1 × 10^6^ HCC cells were suspended in 25 µL serum-free DMEM mixed with 25 µL Matrigel (1 : 1, v/v) and orthotopically injected into the left hepatic lobe of each mouse. After 6~8 weeks, all mice were sacrificed, and the liver (including the xenografted tumors) weight was measured. For histological analysis, liver and lung from mice were collected, fixed, paraffin-embedded, sectioned, stained with hematoxylin and eosin (H&E) and mounted to observe for and analyze the presence of micrometastatic nodules.

### RNA-sequencing analysis (RNA-Seq)

Total RNA from the stable negative control (NC) and TCF12 knockdown (shTCF12) groups of Hep3B cells was extracted using TRIzol reagent (Invitrogen, California, USA) according to the manufacturer's protocol. The total RNA concentration and purity were checked by a NanoDrop 2000 Nucleic Acid and Protein Analyzer (Thermo Scientific, MA, USA). RNA-Seq analysis was performed by Shanghai Biotechnology Corporation (Shanghai, China). The raw data for RNA-Seq have been deposited in the GEO with accession number: GSE133553.

### Dual-luciferase reporter assay

Cells were seeded into 96-well plates. After they were incubated for 24 h, they were cotransfected with the CXCR4 promoters, pWPXL or TCF12 plasmids and internal control PRL-TK reporter plasmids using Lipofectamine^TM^ 2000 (Invitrogen, Carlsbad, CA, USA). After 48 h of culture, the luciferase activities of the cells were measured using the dual-luciferase reporter gene assay system (Promega, USA) according to the manufacturer's protocol. Each transfection condition was performed in triplicate.

### Chromatin immunoprecipitation (Ch-IP) and ChIP-seq assay

The Ch-IP assay was performed according to the manufacturer's protocol (Millipore, Massachusetts, USA). In brief, Hep3B and Li7 cells were crosslinked by formaldehyde, after which then glycine was added to quench unreacted formaldehyde. Cells from each dish were scraped into 1× PBS and centrifuged at 800 × *g* for 5 min. The pellets were resuspended in SDS lysis buffer for 15 min on ice, centrifuged at 800 × *g* for 5 min, and resuspended in nuclear lysis buffer. After the resulting suspension was sonicated, TCF12 antibody (CST11825, Danvers, MA, USA) and protein A magnetic beads were incubated with the lysate overnight at 4 °C and then reverse crosslinked. DNA purification and PCR analysis were performed with the primers listed in Table S1. And a ChIP-seq analysis was further performed by Sinotech Genomics Co., Ltd. (Shanghai, China). The raw data for ChIP-Seq have been deposited in the GEO with accession number: GSE133675.

### Flow cytometry analysis

Flow cytometry analysis was used to determine the cell cycle distribution after TCF12 overexpression and knockdown. In briefly, 1 × 106 cells were plated into 6-well culture plates. The cells were treated with 2 mM thymidine for 24 h to be synchronized at the G1/S boundary. Then, cells were harvested by trypsin after releasing for 0, 12, and 24 h, and washed with 1× PBS twice, fixed with 70% ethanol at -20 °C overnight. Before flow cytometry analysis, cells were washed with 1× PBS and resuspended with 400 mg/mL of propidium iodide, 10 mg/mL of RNase (Sigma-Aldrich, St. Louis, USA), and 0.1% Triton X-100 in 200 µL 1× PBS at 4 °C avoiding light for 30 min. DNA content was quantified using Modfit 3.2 software. Flow cytometry analysis for the population of CXCR4^+^ cells within the total HCC cells was performed as followings: HCC cells (1 × 10^6^ cells/well) were plated into 6-well culture plates and incubated at 37 °C overnight. The cells were washed with PBS twice and resuspended in 100 µL of cell staining buffer. The cells were then blocked with 5 µL of FcR blocking reagent (Biolegend, San Diego, CA, USA) for 5~10 min at room temperature and labeled directly with APC-conjugated anti-human CXCR4 antibody (Biolegend 306510, San Diego, CA, USA) for 30 min at 37 °C. Next, the cells were washed and resuspended in medium supplemented with 0.5% bovine serum albumin (BSA) (Genview, China) for flow cytometry analysis.

### Immunohistochemical (IHC) staining

The clinical-pathological indexes of the human primary HCC samples used in this study were summarized in Table S7 and S8. Paraffin-embedded tissue microarray (TMA) sections were dewaxed in xylene and rehydrated in graded alcohol, followed by routine IHC staining process. Briefly, endogenous peroxidase activity was quenched, 0.1 M citrate buffer (pH 6.0) was boiled and added to the sections for antigen retrieval, nonspecific binding was blocked by treatment with goat serum, primary antibody was incubated with the sections followed by incubation with HRP-conjugated secondary antibody (DAKO EnVision™ Detection Kit, Peroxidase/DAB) for TCF12 and CXCR4 proteins, HRP-anti-Rat IgG (Sigma-Aldrich, A5795) for CD34 protein and the antigen-antibody reaction was visualized with substrate diaminobenzidine (DAB; DAKO, Carpenteria, CA) as the chromogen, the nuclei were counterstained with hematoxylin solution and then cleared in alcohol and xylene. All TMA slides were photographed using a Leica SCN400 slide scanner (Meyer Instruments, Houston, TX, USA) and scored based on the positive percentage and staining intensity of positively staining cells. The staining intensity was defined as 0 and 1, denoting the low expression group and the high expression group, respectively. The antibodies used are shown in Table S3.

### Statistical analysis

Statistical analysis was performed with SPSS version 16.0 (SPSS, Inc., Chicago, IL, USA). The results from at least three independent experiments are presented as the mean ± standard deviation (S.D.). The differences between two groups were determined by two-tailed Student's *t*-test. The correlation between TCF12 expression and CXCR4 or CXCL12 expression in human primary HCC tissues was determined using Pearson's coefficient tests. Chi-square test or Fisher's exact test was used to assess the correlations between TCF12 and clinicopathological features. The survival analysis was analyzed using the Kaplan-Meier method and log-rank test. *P* < 0.05 was considered statistically significant.

## Results

### TCF12 promotes cell proliferation and induces G0/G1 to S phase transition in HCC cells

**Figure 1:**
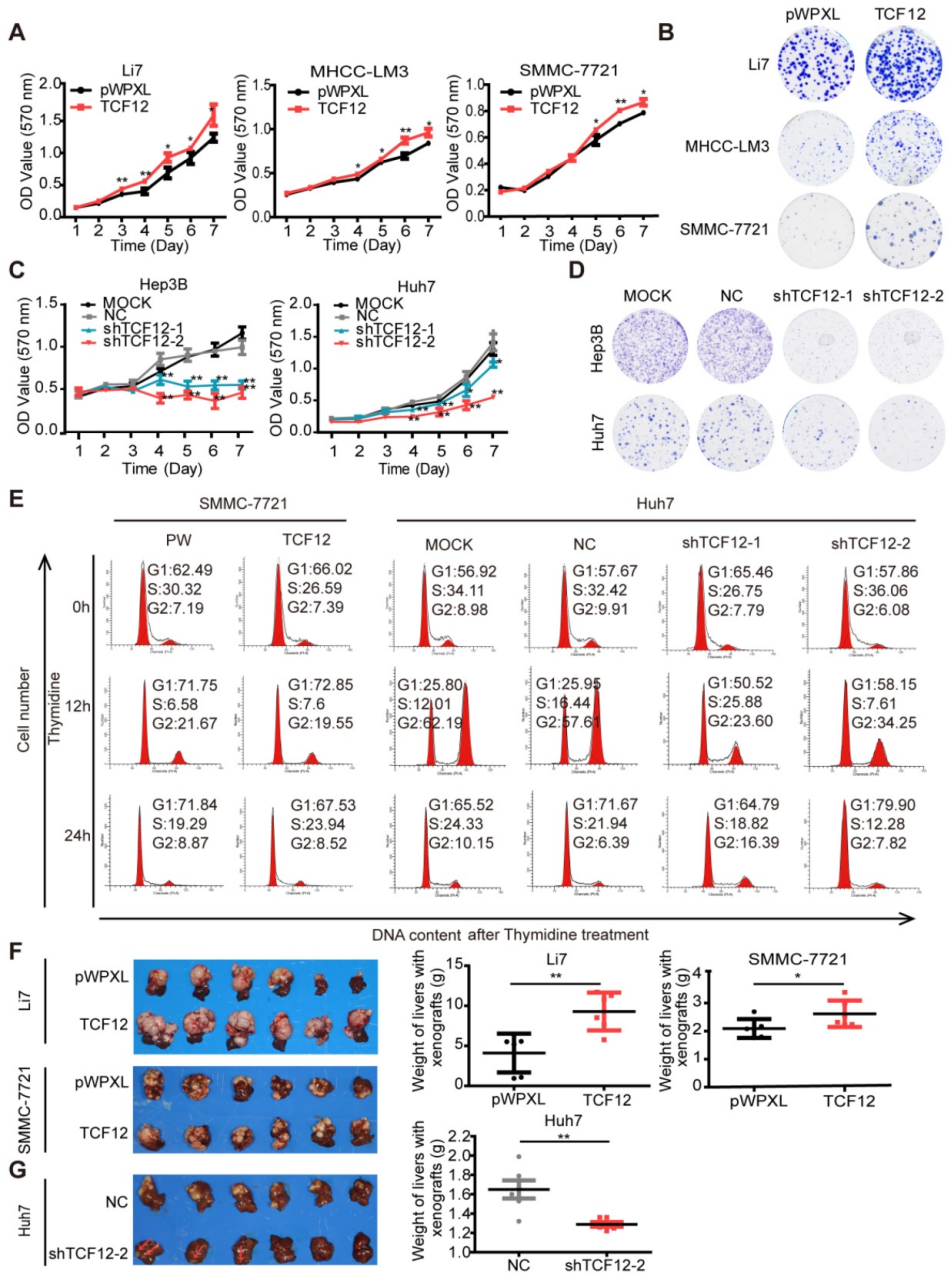
** TCF12 promotes HCC cell proliferation *in vitro* and tumorigenicity *in vivo*. (A, C)** Growth curve of HCC cells with TCF12 overexpression or knockdown was determined by performing the MTT assay for 7 days. pWPXL, empty vector; MOCK, blank control; NC: negative control. **(B, D)** Representative images of the colony formation of HCC cells with TCF12 overexpression or knockdown. **(E)** The cell cycle distribution of SMMC-7721 cells with TCF12 overexpression and Huh7 cells with TCF12 knockdown collected at 0 h, 12 h and 24 h after synchronizing with 2 mM thymidine. **(F, G)** TCF12 overexpression increased the growth of Li7- and SMMC-7221-derived orthotopic xenografts in nude mice, whereas TCF12 knockdown in Huh7 cells had the opposite effect. The red arrow indicates the tumor nodules in livers. Liver weights with xenografted tumors are shown in the right panels. Data are shown as the mean ± S.D. *P < 0.05, **P < 0.01.

To explore the biological function of TCF12 in HCC, endogenous TCF12 expression levels in a set of hepatoma cell lines and L02 cells were initially measured by RT-qPCR and western blot; the data revealed that TCF12 was strongly expressed in Hep3B and Huh7 cells; moderately expressed in MHCC- LM3, MHCC-97H, PLC/PRF/5, SK-Hep 1 and Li7 cells; and weakly expressed in MHCC-97L, SMMC- 7721 and L02 cells (Figure S1A). We successfully established stable ectopic TCF12 expression in Li7, MHCC-LM3 and SMMC-7721 cells and stable knockdown of TCF12 in Hep3B and Huh7 cells (Figure S1B). The MTT assay and colony formation assay indicated that ectopic TCF12 expression promoted cell proliferation and colony formation in Li7, MHCC-LM3 and SMMC-7721 cells (Figures 1A, 1B and S1C), whereas TCF12 knockdown inhibited cell proliferation and colony formation in Hep3B and Huh7 cells (Figures 1C, 1D and S1D). Next, we performed a cell cycle analysis to investigate whether TCF12 accelerated HCC cell proliferation via alteration of the cell cycle. The results showed that TCF12 knockdown in Huh7 cells led to a G1/S arrest, together with a remarkable decline of the cell population in S phase. While, it's not obvious after TCF12 overexpression in SMMC-7721 cells (Figure S1E). Then we further treated 2 mM thymidine for 24 h to synchronize cells at the G1/S phase border. After releasing, flow cytometry analysis showed that the percentage of cells at G1 phase was significantly higher after TCF12 knockdown in Huh7 cells and was lower in TCF12-overexpressing SMMC-7721 cells when compared with their control (Figure 1E and Table S4). The expression levels of CDK4, cyclinD1/ D2, cyclin E1 were increased in TCF12-overexpressing cells and decreased in TCF12-knockdown cells, whereas the expression of CDK2 and CDK6 did not change with TCF12 alternation (Figure S1F). So, all results suggest that TCF12 promotes cell proliferation through inducing G1/S transition in HCC cells.

We also used an orthotopic mouse xenograft model to verify whether TCF12 affected the tumorigenesis of HCC cells *in vivo*. Quantification of tumor weight (liver with xenograft) showed that Li7 and SMMC-7721 cells overexpressing TCF12 generated larger tumor masses than did the corresponding control cells (Figure 1F), and the xenograft tissues maintained high expression levels of TCF12 in the TCF12-overexpressing group (Figures S1G). Conversely, tumor growth was obviously decreased in mice injected with Huh7 cells with stable TCF12 knockdown (Huh7-shTCF12-2) compared to that of mice in the negative control (NC) group. Tumor masses formed in the livers of all six mice in the NC group, whereas only one large and two small tumor nodules were observed in the shTCF12 group (Figure 1G). Collectively, these results indicate that TCF12 has an oncogenic function and promotes tumorigenesis of HCC *in vitro* and* in vivo*.

### TCF12 promotes HCC cell migration, invasion *in vitro* and lung metastasis *in vivo*

**Figure 2:**
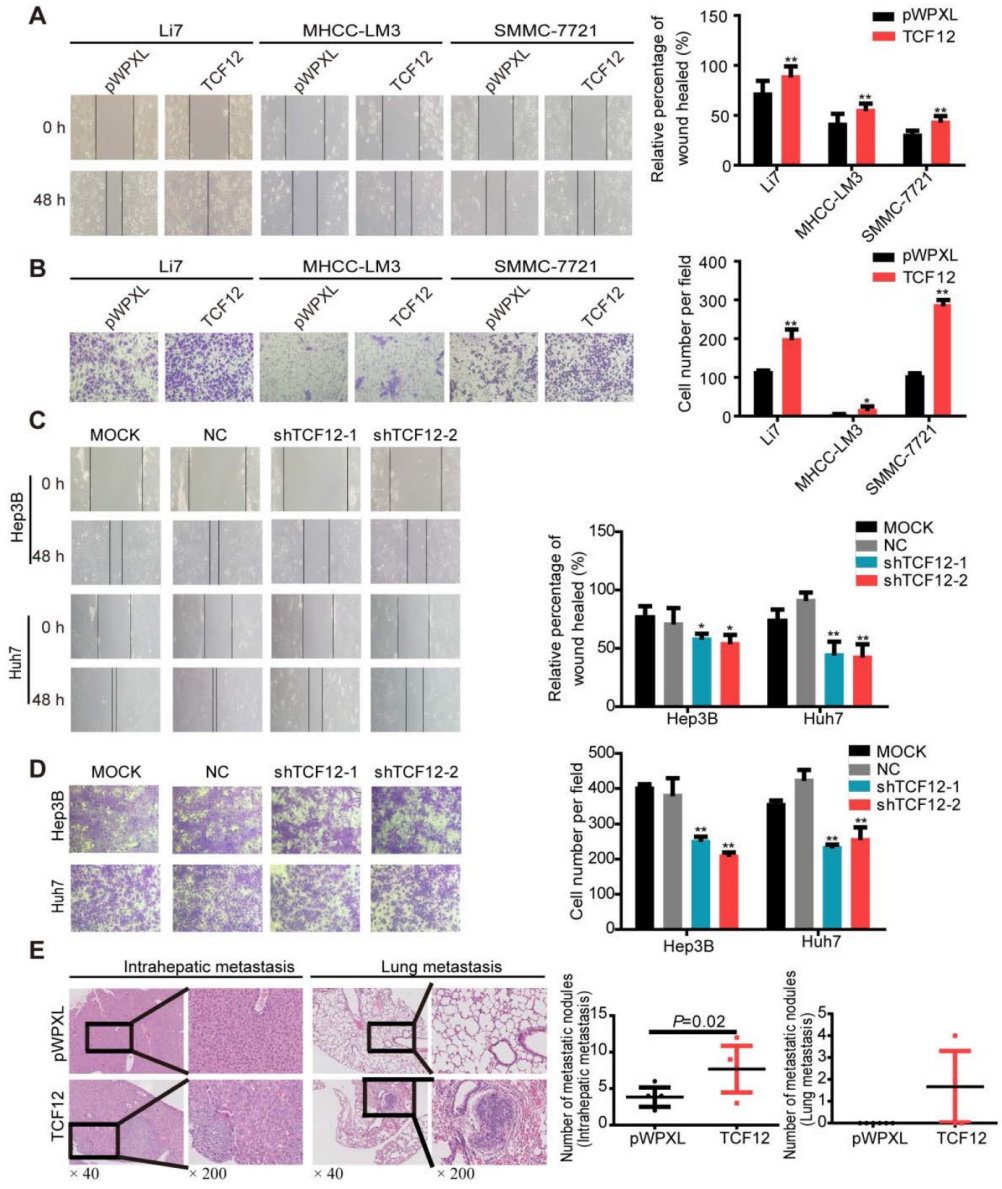
** TCF12 promotes HCC cell mobility *in vitro* and lung metastasis *in vivo*. (A, B)** Overexpression of TCF12 increased HCC cell migration and invasion *in vitro* as indicated by the wound healing and invasion assays, respectively. Representative micrographs are shown in the left panels. The bar graph represents quantitative analysis data with three replicates (right panels). **(C, D)** Silencing TCF12 suppressed HCC cell migration and invasion *in vitro* as indicated by the wound healing and invasion assays, respectively. Representative images are shown in the left panels. The bar graph indicates quantitative analysis data with three replicates (right panels). **(E)** Representative H&E pictures of intrahepatic metastatic and lung metastatic nodules of the pWPXL and TCF12 overexpression groups (original magnification of left images, ×40; right enlarged images, ×200). The numbers of intrahepatic metastatic and lung metastatic nodules are presented in the left panels (bar graph) (n=6/group). Data are shown as the mean ± S.D. *P < 0.05, **P < 0.01.

Previous documents suggested a close correlation between TCF12 expression and tumor metastasis 14-17. We thus investigated whether TCF12 in HCC affected cell migration and invasion. *In vitro* wound healing and Matrigel invasion assays showed that TCF12 overexpression increased the migration and invasion of HCC cells (Figures 2A and 2B), whereas TCF12 knockdown repressed HCC cell migration and invasion *in vitro* (Figures 2C and 2D). The paraffin sections of liver and lung tissues from the orthotopic mouse xenograft model were prepared, and H&E staining showed that mice injected with SMMC-7721 cells with TCF12 overexpression had more intrahepatic and lung metastatic nodules than control mice (Figure 2E and Table S5). Collectively, these findings indicate that TCF12 promotes HCC metastasis *in vitro* and *in vivo*.

Since epithelial-mesenchymal transition (EMT) is a crucial event in the progression of tumor metastasis 28, we further investigate whether TCF12 regulated the EMT markers of HCC cells by western blot. As shown in Figure S2A, TCF12 overexpression in Li7 and SMMC-7721 cells led to decreased expression of epithelial marker E-cadherin, whereas increased expression of mesenchymal markers N-cadherin, as well as the essential EMT transcription factors Snail and Slug. Conversely, TCF12 knockdown in Hep3B and Huh7 cells decreased the expression of N-cadherin, Snail and Slug, and was accompanied by increased expression of E-cadherin (Figure S2B). Consistent with the expression of EMT-related markers in HCC cells, western blot also confirmed that TCF12 overexpression promoted the EMT in xenograft tissues formed by SMCC-7721 cells (Figure S2C). Therefore, these results reveal that TCF12 induces EMT in HCC cells.

### CXCR4 is a direct downstream target gene of TCF12

To elucidate the key molecules participating in TCF12-induced proliferation and metastasis of HCC cells, we examined the transcriptome of Hep3B-NC and Hep3B-shTCF12-2 cells by RNA-Seq analysis. We further performed gene ontology enrichment analysis and found that these genes were significantly associated with cancer related functions, including cell cycle regulation, cell migration, cell apoptosis and so on (Figure S3A). Subsequently, we screened a set of HCC-related target genes and further detected with RT-qPCR (Table S6). RT-qPCR verified that TCF12 knockdown significantly decreased CXCR4 and increased CD226 expression (expression ratio showing greater than 2.0-fold or less than 0.5-fold difference compared with the control group), which showed an opposite trend in TCF12-overexpressing Li7 cells (Figure S3B). Given the critical role of CXCR4 in HCC progression 20, 22, 25, 29 and the most obvious expression-altering gene in TCF12 knockdown cells, we decided to investigate the role and the underlying mechanism of CXCR4 in pro-tumor function of TCF12 in HCC.

**Figure 3:**
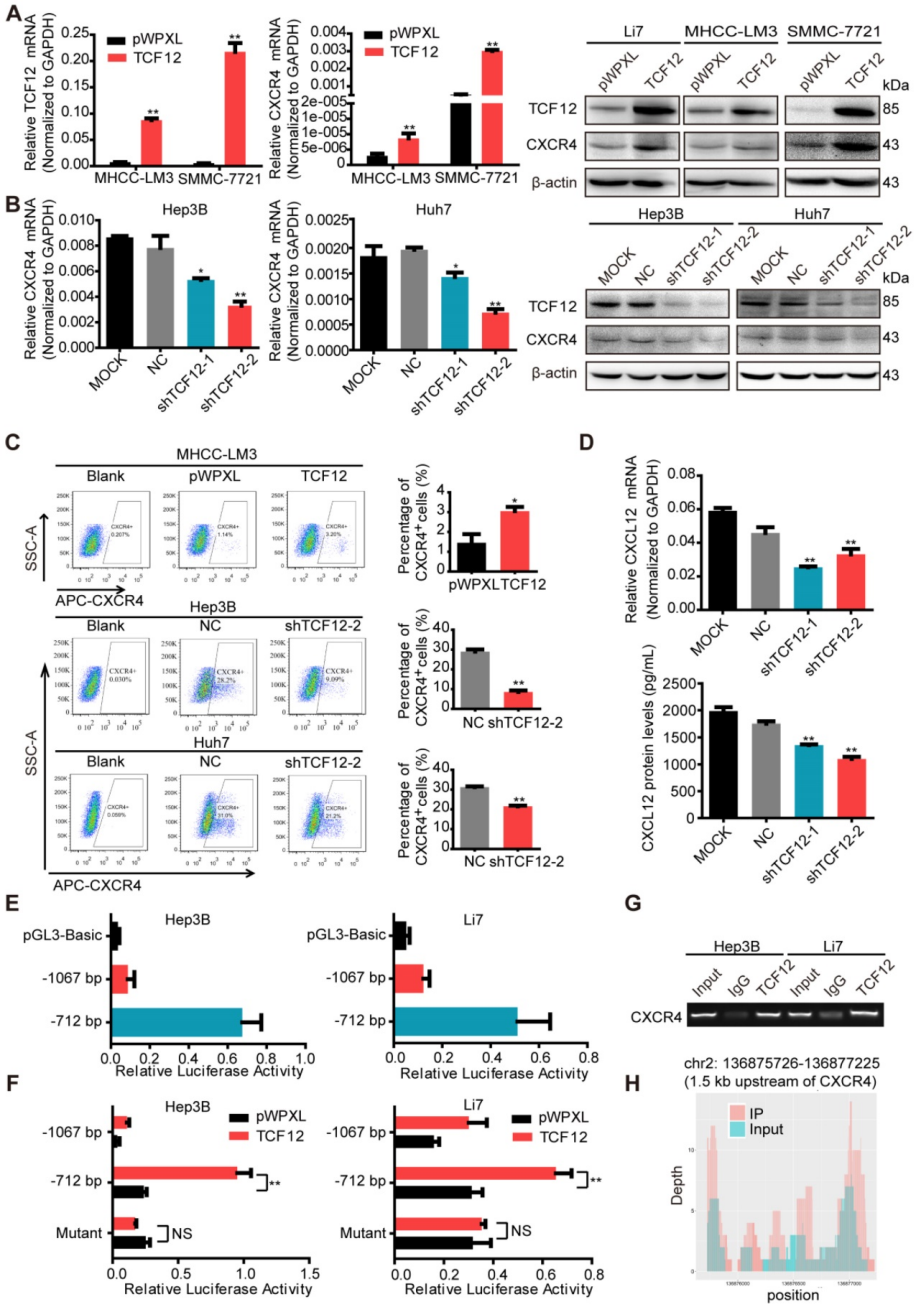
** TCF12 regulates CXCR4 and CXCL12 expression in HCC. (A)** The mRNA and protein levels of CXCR4 in HCC cells expressing pWPXL empty vector or TCF12 were respectively detected by RT-qPCR (left panels) and western blot (right panels). **(B)** The mRNA and protein levels of CXCR4 in Hep3B and Huh7 cells with TCF12 knockdown were respectively detected by RT-qPCR (left panels) and western blot (right panels). **(C)** The percentage of CXCR4^+^ cells among the tested HCC cell lines with TCF12 overexpression (MHCC-LM3) or knockdown (Hep3B and Huh7) was detected by flow cytometry analysis. The bar graph shows quantitative analysis data with three replicates (right panels). **(D)** RT-qPCR was used to detect CXCL12 mRNA expression in Hep3B cells with TCF12 knockdown (upper panel). And ELISA was performed to measure the CXCL12 protein level in culture medium from TCF12-silenced Hep3B cells (lower panel). **(E)** The relative luciferase activities of the CXCR4 promoter and the truncated construct (-712 bp) upon transfection in Hep3B and Li7 cells. **(F)** The relative luciferase activities of the CXCR4 promoter, the truncated construct (-712 bp) and the mutant constructs of the CXCR4 promoter after transfection with pWPXL empty vector or TCF12 in Hep3B and Li7 cells. **(G)** Agarose electrophoresis for Ch-IP analysis of TCF12 directly binding to the CXCR4 promoter region in Hep3B and Li7 cells. Rabbit IgG was used as a negative control.** (H)** Peak signals detected from the ChIP sequecing revealed that CXCR4 might be a potential target of TCF12. Data are shown as the mean ± S.D. *P < 0.05, **P < 0.01, NS: no significance.

RT-qPCR and western blot were then performed to detect CXCR4 expression in other HCC cells with TCF12 overexpression or silencing; the results further supported CXCR4 as a potential downstream target gene of TCF12 (Figures 3A, 3B and S3C). Moreover, overexpression of TCF12 in two immortalized normal liver hepatocyte cell lines (L02 and MIHA) could also up-regulate expression of CXCR4 (Figure S3D). It was previously reported that CXCR4 was trapped in the cytoplasm of the majority of HCC cells, which resulted in negligible responses to CXCL12 30. However, the results of the flow cytometry analysis in our study revealed that the cell surface expression of CXCR4 was present in Li7, MHCC-LM3, SMMC-7721, Hep3B and Huh7 cells, with high expression in Li7 and SMMC-7721 cells, moderate expression in Hep3B and Huh7 cells and low expression in MHCC-LM3 cells (Figure S3E). In addition, the population of CXCR4^+^ cells within the total HCC cells cultures was increased by TCF12 overexpression in MHCC-LM3 cells and decreased by TCF12 silencing in Hep3B and Huh7 cells (Figure 3C). Usually, the specific ligand of the CXCR4 receptor is CXCL12, and the CXCL12/ CXCR4 signaling axis plays a critical role in the progression and metastasis of HCC. To explore whether TCF12 can also increase CXCL12 expression and secretion in HCC cells, we first detected CXCL12 mRNA expression in 24 HCC cell lines using RT-qPCR and found that CXCL12 could be detected in 12 HCC cell lines, with TCF12 mRNA highly expressed in most HCC cells (Figure S3F). Meanwhile, CXCL12 protein secretion from the five HCC cell lines used in our study was detected by ELISA. We found that CXCL12 was highly expressed in Hep3B cells but weakly detectable in the other four HCC cells (Figure S3G), and the inhibition of TCF12 expression in Hep3B cells led to decreased mRNA expression and protein secretion of CXCL12 (Fig 3D).

To further explore whether CXCR4 is a direct target of TCF12, the JASPAR database (http://jaspar. genereg.net/) was used to analyze the potential TCF12 binding sites on the CXCR4 promoter, mainly located at -989 ~ -999 bp, -878 ~ -888 bp and -626 ~ -636 bp relative to the TSS (Figure S3H). Then, we constructed a luciferase reporter plasmid with a 1280 bp CXCR4 promoter region (-1067 ~ +213 bp relative to the TSS) and a truncated clone of the region (-712 ~ +213 bp relative to the TSS). The promoter activity of the constructs all showed higher luciferase activity than that of the pGL3 vector in Hep3B and Li7 cells and was significantly induced by overexpression of TCF12, mainly at the region of -715 ~ +213 bp (Figures 3E and 3F). Mutant constructs for TCF12 binding sites (-626 ~ -636 bp) were generated using site-specific mutagenesis (Figure S3I). The enhanced luciferase activity was reversed by transfection with the mutant promoter region (Figure 3F). The Ch-IP assay and CHIP-seq data further confirmed that TCF12 directly binds to the promoter of the CXCR4 gene in HCC cells (Figures 3G and 3H). Taken together, these data suggest that CXCR4 is a direct transcriptional target of TCF12.

### CXCR4 is essential for TCF12-induced cell proliferation, migration and invasion *in vitro*

**Figure 4:**
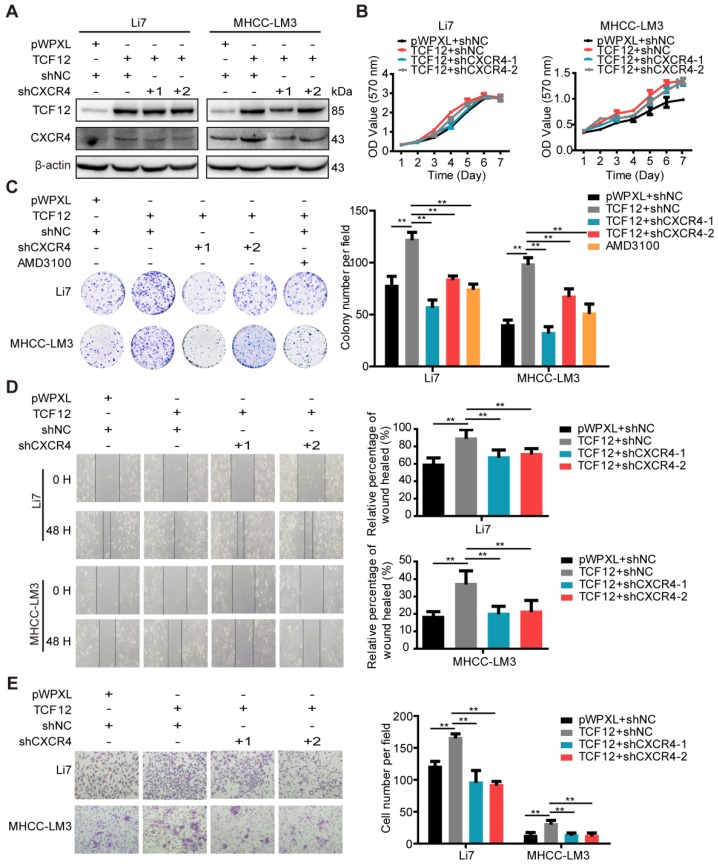
** CXCR4 silencing inhibits the pro-oncogenic capacity induced by TCF12 overexpression in HCC cells. (A)** Li7 and MHCC-LM3 cells that stably expressed TCF12 were transfected with either shNC or shRNA targeting CXCR4, and the protein knockdown efficiency of CXCR4 was detected by western blot. **(B)** The influence of silencing CXCR4 on the effects of TCF12-induced growth in Li7 and MHCC-LM3 cells was measured by the MTT assay. **(C)** The influence of silencing CXCR4 or treatment with the CXCR4 antagonist AMD3100 (100 nM) on the effects of TCF12-induced colony formation in Li7 and MHCC-LM3 cells was measured by an *in vitro* colony formation assay. **(D, E)** *In vitro* wound healing and transwell invasion assays showed that silencing CXCR4 could abrogate the effects of TCF12 overexpression on the migration **(D)** and invasion **(E)**, respectively, of HCC cells. Representative micrographs are shown in the left panels. The bar graph in the right panels shows quantitative analysis data with three replicates. Data are shown as the mean ± S.D. **P < 0.01.

Numerous studies have demonstrated correlations between high CXCR4 expression and aggressive tumor behavior as well as poor prognosis of HCC 20, 22, 25, 29. Therefore, we investigated whether TCF12-induced enhancement of cell proliferation and metastasis was CXCR4-dependent. TCF12-overexpressing cells were transfected with shCXCR4 to silence CXCR4 expression. Our results indicate that CXCR4 knockdown could reverse TCF12-induced CXCR4 upregulation in TCF12-overexpressing HCC cells (Figures 4A and S4A-S4C). In addition, MTT and colony formation assays revealed that CXCR4 knockdown significantly reduced TCF12-induced cell proliferation (Figure 4B and 4C). As expected, AMD3100 (a CXCR4 antagonist) also attenuated TCF12-induced cell proliferation (Figure 4C). Moreover, CXCR4 knockdown also reversed TCF12-induced cell proliferation in MIHA cells (Figure S4D). In addition, *in vitro* wound healing and transwell assays showed that the migration and invasion capacities of TCF12-overexpressing HCC cells were decreased by CXCR4 knockdown (Figures 4D, 4E, S4E and S4F). These results suggest that CXCR4 is essential for the TCF12-mediated effects on cell growth, migration and invasion of HCC cells.

### CXCL12/CXCR4 mediates the effects of TCF12 on angiogenesis *in vitro* and *in vivo*

**Figure 5:**
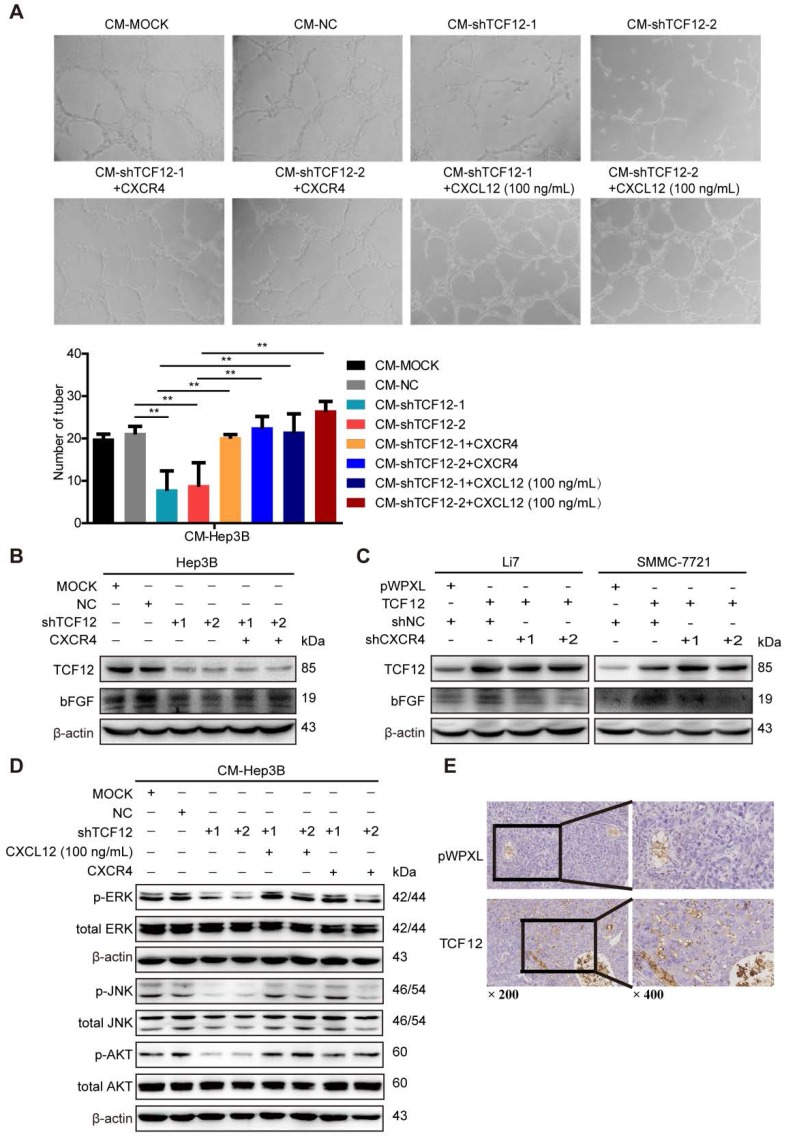
** The CXCL12-CXCR4 axis participates in the effects of TCF12 on angiogenesis *in vitro* and *in vivo*. (A)** Representative micrographs (original magnification, × 20) of the tube formation assay of HUVECs after treatment for 8 h with CM from the indicated groups of Hep3B cells. Quantitative analyses of the HUVEC cell tube formation assay described in the left lower panel. Data are shown as the mean ± S.D. from experiments with three replicates. **P < 0.01. **(B)** Western blot analysis of the levels of bFGF proteins in TCF12-silenced Hep3B cells overexpressing CXCR4. **(C)** Western blot analysis of the levels of bFGF in TCF12 overexpressing Li7 and SMMC-7721 cells transfected with shNC or shCXCR4. **(D)** Western blot analysis of the levels of phosphorylated ERK, JNK and AKT protein in HUVECs treated with CM from blank control (MOCK), Hep3B cells transfected with shNC or shTCF12, or Hep3B-shTCF12 cells with CXCL12 (100 ng/mL) stimulation or CXCR4 overexpression. **(E)** Immunohistochemical staining for CD34 was performed in tissue sections from orthotopic mouse xenografts derived from SMMC-7721 (original magnification of left images, × 200; right enlarged images, × 400).

A marked association between the CXCL12-CXCR4 axis and angiogenesis in various human tumors has been reported previously and CXCL12 has been shown to act as direct inducers of angiogenesis 23-25, 31, 32. As TCF12 regulated CXCR4 and CXCL12 expression, an *in vitro* HUVECs tube formation assay was conducted to explore whether TCF12 plays a functional role in tumor angiogenesis. The results showed a remarkable decrease in tubular structure formation in HUVECs cultured with CM from Hep3B cells with TCF12 knockdown (CM-shTCF12) compared with that in HUVECs cultured with control CM (CM-MOCK, CM-NC), but increases were observed after stimulation with recombinant human CXCL12 protein (100 ng/mL) (Figure 5A). Moreover, the proliferation of HUVECs was unaffected by the above mentioned treatments (Figure S5A); however, the migration of HUVECs was remarkably decreased after treatment with CM-shTCF12 (Figure S5B). Meanwhile, we observed that the tube formation potential was also enhanced after CXCR4 overexpression in TCF12 knockdown cells (Figure 5A). To further elucidate the molecular mechanism of CXCR4-mediated angiogenesis induced by TCF12, the 10 kinds of common angiogenic factors were detected using RT-qPCR. The results showed that the mRNA level of basic fibroblast growth factor (bFGF) was increased about 2-fold both in TCF12 overexpressing Li7 and SMMC-7721 cells, decreased 3.4-fold in TCF12 knocking down Hep3B cells (Figure S5C-S5E). Further western blot showed knocking down TCF12 in Hep3B induced a decrease in the protein level of bFGF compared with their expression in the control cells and reversed by CXCR4 overexpression in TCF12-knockdown cells (Figure 5B). In contrast, a higher level of bFGF in the TCF12 overexpressing Li7 and SMMC-7721 cells than in the control cells, which was reduced by CXCR4 knockdown in TCF12 overexpressing HCC cells (Figure 5C). Moreover, western blot of HUVECs showed a clear decrease in p-ERK, p-JNK and p-AKT levels after treatment with CM-shTCF12 which were rescued after treatment for 30 min with CXCL12 protein (100 ng/mL) or CXCR4 overexpression (Figure 5D). In addition, we observed that compared with the control group, xenografts formed by SMMC-7721 overexpressed with TCF12 exhibited more vascular density (Figure 5E). These results suggest that the involvement of TCF12 in the angiogenesis of HUVECs could be mediated by the CXCL12-CXCR4 axis, which activates its downstream effectors MAPK/ERK/JNK and AKT in HUVECs to initiate cell migration and angiogenesis.

### CXCL12/CXCR4 mediates the effects of TCF12 on chemotaxis of HCC cells toward CXCL12

**Figure 6:**
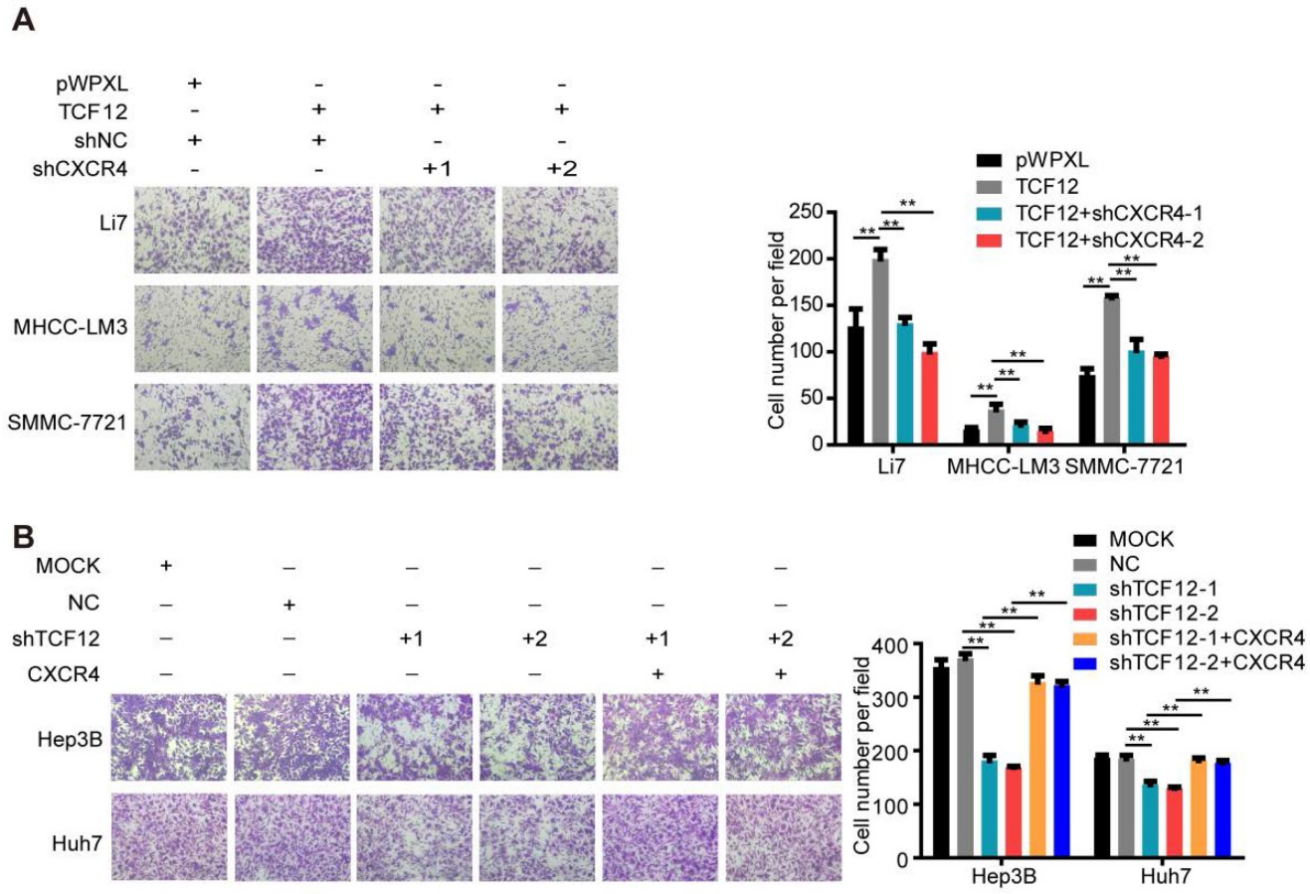
** The CXCL12-CXCR4 axis participates in the effects of TCF12 on HCC cells chemotaxis *in vitro***.** (A)** CXCR4 knockdown decreased TCF12-induced chemotaxis of Li7 and MHCC-LM3 cells toward CXCL12 stimulation (100 ng/mL). The right panel shows the counts for invaded cells.** (B)** CXCR4 overexpression reversed the inhibitory effects of TCF12 silencing on the chemotaxis of Hep3B and Huh7 cells. The right panel shows the counts of invaded cells. Data are shown as the mean ± S.D. from experiments with three replicates. **P < 0.01.

The role of CXCL12/CXCR4 on chemotaxis in various cancers is vital to the development of metastases, as evidenced by CXCR4-expressing cancer cells migrating *via* chemotaxis through a CXCL12 gradient to form metastatic lesions 33-35. Thus, we further explored the effect of TCF12 on the chemotaxis of HCC cells toward high concentrations of CXCL12 using an *in vitro* Matrigel invasion assay. Ectopic TCF12 expression promoted the chemotaxis of Li7, MHCC-LM3 and SMMC-7721 cells in response to recombinant human CXCL12 protein (100 ng/mL), but this activity was abolished by CXCR4 knockdown (Figure 6A). Conversely, silencing TCF12 in Huh7 and Hep3B cells suppressed the chemotaxis of HCC cells, which was increased upon CXCR4 overexpression (Figure 6B). Quality control experiments to prove successful stable overexpression of CXCR4 in Hep3B and Huh7 cells with TCF12 knockdown are demonstrated in Figure S6. These results suggest that TCF12 can enhance the chemotaxis ability of HCC cells in the tumor microenvironment.

### TCF12-induced upregulation of CXCR4 activates the MAPK/ERK and PI3K/AKT signaling pathways

**Figure 7:**
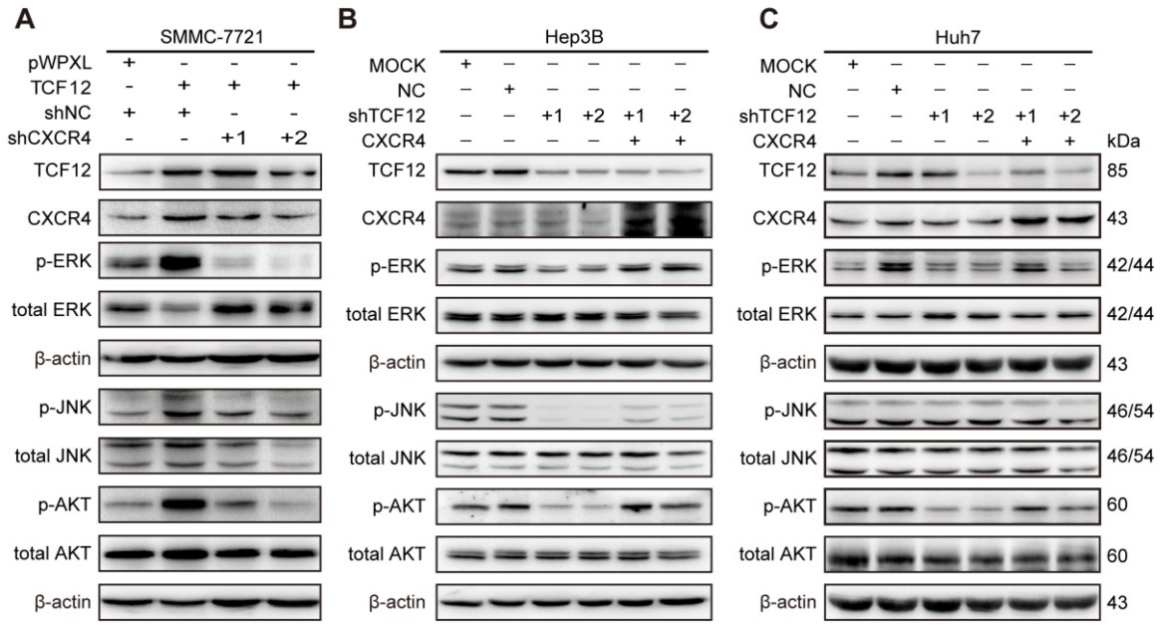
** TCF12-induced CXCR4 upregulation activates the MAPK/ERK and PI3K/AKT signaling pathways. (A)** Western blot analysis of the levels of phosphorylated ERK, AKT and JNK in TCF12 overexpressing SMMC-7721 cells transfected with shNC or shCXCR4.** (B, C)** Western blot analysis of the levels of phosphorylated ERK, AKT and JNK proteins in TCF12-silenced cells overexpressing CXCR4.

Based on the findings that CXCL12-CXCR4 axis-dependent cell proliferation and metastasis are correlated with activation of the ERK1/2 and AKT pathways 36, 37, western blotting was carried out. The results showed that p-ERK, p-JNK and p-AKT levels were increased after TCF12 overexpression in Li7, MHCC-LM3 and SMMC-7721 cells and were decreased after TCF12 knockdown in Hep3B cells (Figures S7A and S7B). Further study showed that CXCR4 knockdown could reverse the level of phosphorylated ERK, JNK and AKT protein upregulated by TCF12 (Figure 7A), whereas CXCR4 overexpression in Hep3B cells with TCF12 knockdown could restore the level of phosphorylated ERK, JNK and AKT protein (Figure 7B). The levels of phosphorylated ERK and AKT were also decreased in Huh7 cells after TCF12 knockdown but were restored by CXCR4 overexpression, while JNK phosphorylation was unaffected (Figure 7C), suggesting that the MAPK/JNK pathway is not a common pathway activated by TCF12 in HCC cell lines. However ERK and AKT phosphorylation levels restored weakly in Hep3B cells and remained unchanged in Huh7 cell in cultures exposed to recombinant CXCL12 protein (100 ng/mL), indicating that the receptor- rather than ligand-dependent role of TCF12 in MAPK/ERK and PI3K/AKT activation (Figures S7C). These data suggest that TCF12 enhances the transcriptional activation of CXCR4, thereby activating the MAPK/ERK and PI3K/AKT signaling pathways.

### TCF12 expression is positively correlated with CXCR4 and CXCL12 expression and a poor prognosis in HCC patients

**Figure 8:**
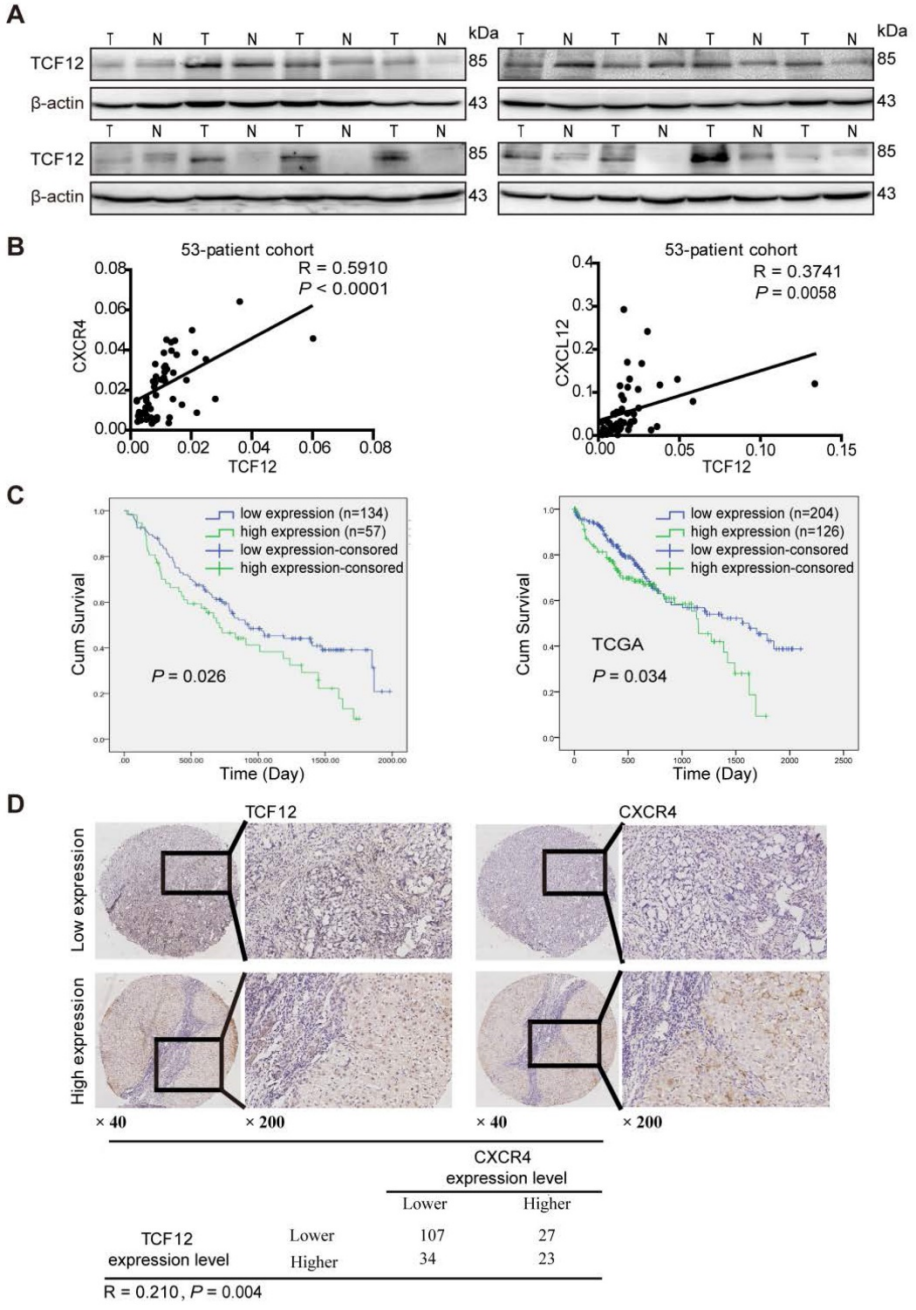
** TCF12 expression is positively correlated with both CXCR4 and CXCL12 expression in primary HCC tissues and the poor survival of patients. (A)** Western blot was used to detect TCF12 protein expression in 16 paired HCC tissues and noncancerous liver tissues. **(B)** Scatter plots showing the correlation between TCF12 mRNA and either CXCR4 or CXCL12 mRNA in 53 HCC tissues from primary HCC patients (R, Pearson correlation coefficient; *P*, p value). **(C)** Kaplan-Meier analysis of OS in patients with low expression or high expression of TCF12; 191 HCC patients (left panel) and 330 HCC patients from the TCGA database (right panel) were assessed. *P* value was determined by the log-rank test. **(D)** Representative IHC staining of TCF12 and CXCR4 in 191 human HCC tissues. The positive correlation between the TCF12 and CXCR4 IHC scores is presented in the lower panel.

Western blot analysis showed that TCF12 was upregulated in HCC tissues (Figure 8A). Further the TCGA database analysis showed that TCF12 expression was upregulated in HCC tissues; and there was no statistical significance between the mRNA expression levels of CXCR4 in the HCC tissues and those in the paired adjacent liver tissues, and the expression levels of CXCL12 in the cancer tissues were significantly lower than those in the paired adjacent liver tissues (Figure S8A).

To further investigate the relationships of TCF12, CXCL12 and CXCR4 in human primary HCC tissues, RT-qPCR was used to detect the mRNA expression levels of TCF12, CXCR4 and CXCL12 in HCC tissues. As shown in Figure 8B, TCF12 mRNA expression significantly correlated with CXCR4 (R=0.5910, *P*<0.0001) and CXCL12 (R=0.3741, *P*=0.0058) mRNA expression, which was in accordance with the analysis in the TCGA cohort (Figure S8B). Western blotting also revealed a positive association between TCF12 and CXCR4 protein levels in HCC patients (R=0.3782, *P*=0.03) (Figure S8C).

Subsequently, we evaluated TCF12 and CXCR4 expression in 191 HCC specimens by IHC analysis. The expression intensities were scored as 0 or 1 for low and high expression, respectively. The results showed that TCF12 overexpression was associated with the histological grade (*P*=0.06, Table S7), although the observed difference did not reach statistical significance. Furthermore, CXCR4 expression was significantly related to the histological grade and hepatic cirrhosis (*P*=0.035 and 0.043, respectively, Table S8). In addition, Kaplan-Meier survival analysis revealed that patients with high TCF12 expression showed worse OS than patients with low TCF12 expression in 191 HCC patients (*P*=0.026), which was consistent with the analysis in the TCGA cohort (*P*=0.034, Figure 8C). Last, a significant positive correlation between TCF12 and CXCR4 expression was also observed in 191 HCC samples (R=0.210, *P*=0.004) (Figure 8D).

## Discussion

In this study, we aimed to explore and focus on the significance of TCF12 as an oncogenic transcription factor in HCC. In recent years, emerging evidence has indicated the critical roles of TCF12 in the pathogenesis of multiple tumors. Although TCF12 is an oncogenic molecule in CRC and some other cancers, its function as a tumor suppressor has become more evident in a subset of malignancies. TCF12 plays different roles in various malignancies may lie in the tissue heterogeneity and different cellular pathways involved in different cellular context. To date, the biological function of TCF12 in HCC is not reported yet. We report here a novel finding that TCF12 plays an oncogenic role in HCC, and the expression of TCF12 is a potential prognostic marker for HCC, because TCF12 was found to be overexpressed in human HCC tissues compared with matched noncancerous liver tissues and was correlated with poor survival of HCC patients. Additionally, we revealed that TCF12 binds to the CXCR4 promoter to upregulate CXCR4 expression, which can activate downstream pathways of MAPK/ERK and PI3K/AKT signaling to promote HCC cell proliferation and metastasis. However, in addition to CXCR4, other potential targets could emerge from this study in the future for follow up.

In the current study, our *in vitro* assays showed that TCF12 promoted cell proliferation, migration and invasion of HCC cells. In addition, the results from our HCC xenograft tumor model demonstrated that TCF12 overexpression enhanced tumor formation and lung metastasis, whereas TCF12 knockdown suppressed hepatocarcinogenesis. Furthermore, TCF12 overexpression induced cell cycle progression while knockdown of TCF12 arrested the G0/G1 to S phase transition, which could partially explain the proliferation effect induced by TCF12. Besides, as reported in CRC 14, 15 and GBC 16, TCF12 induced EMT by downregulation of epithelial marker expression and upregulation of mesenchymal markers expression. The above results further confirmed the oncogenic role of TCF12 in HCC.

Subsequently, we identified CXCR4 as a potential downstream target of TCF12 by RNA-Seq analysis. TCF12 directly bound to the CXCR4 promoter and enhanced the transcriptional activation of CXCR4 in HCC cells. As expected, TCF12-induced proliferation, migration and invasion could be reversed by silencing CXCR4. Moreover, TCF12 and CXCR4 expression were also positively correlated in clinical HCC samples, strongly indicating that CXCR4 is likely regulated by TCF12. We also found that TCF12 overexpression could increase CXCR4 expression in normal liver hepatocytes and CXCR4 knockdown reversed TCF12-induced MIHA cells growth, demonstrating that TCF12 could promote HCC tumorigenesis by upregulating CXCR4 expression. It was reported that TCF12 enhanced CXCL12 secretion in CAFs to promote the growth of breast cancer cells 38. We also found that TCF12 knockdown was accompanied by a decrease in CXCL12 expression in Hep3B cells with high CXLC12 expression. E-box motifs were identified in the gene sequence of the CXCL12 promoter on the JASPAR website, and more in-depth experiments are needed to clarify whether TCF12 can regulate CXCL12. Consistent with previous findings 20, 22, 39, our IHC analysis revealed that CXCR4 protein was only detected in HCC tissues but not in paired adjacent noncancerous liver tissues. In HCC, CXCR4 expression has been correlated with tumor size, distant dissemination and poor prognosis of patients 20, 22, 39-41. Here, we observe a correlation between CXCR4 expression and the histological grade and hepatic cirrhosis, but not with tumor size and metastasis in HCC tissues, which could be most likely due to the low sensitivity and specificity of the CXCR4 antibody for IHC and needs to be studied in the future.

The role of CXCL12/CXCR4 on chemotaxis has been reported in various tumors, as shown by the high levels of CXCL12 in bone, lymph nodes and lung which can attract CXCR4-expressing cancer cells 42-45. In this study, an *in vitro* transwell assay showed that the chemotaxis of HCC cells toward high concentrations of CXCL12 was significantly enhanced by TCF12 overexpression and suppressed by TCF12 knockdown, which was further verified by our results showing the role of TCF12 in metastasis *in vivo*.

Emerging evidence has indicated that the CXCL12-CXCR4 axis participates in tumor angiogenesis, including in HCC, through autocrine and paracrine pathways 23-25, 46. Here, we report that TCF12 promoted angiogenesis via the CXCL12- CXCR4 axis. On one hand, TCF12 participated in angiogenesis through the regulation of CXCL12 expression, which has been shown to act as a direct inducer of angiogenesis in HCC. One the other hand, TCF12 overexpression in HCC cells could activate the MAPK/ERK and PI3K/AKT signaling pathways through CXCR4 upregulation, all of which are involved in the angiogenesis. Further studies revealed TCF12 promotes angiogenesis through the CXCR4-induced bFGF expression, a potent and representative angiogenic growth factor involved in HCC development 47, 48.

**Figure 9:**
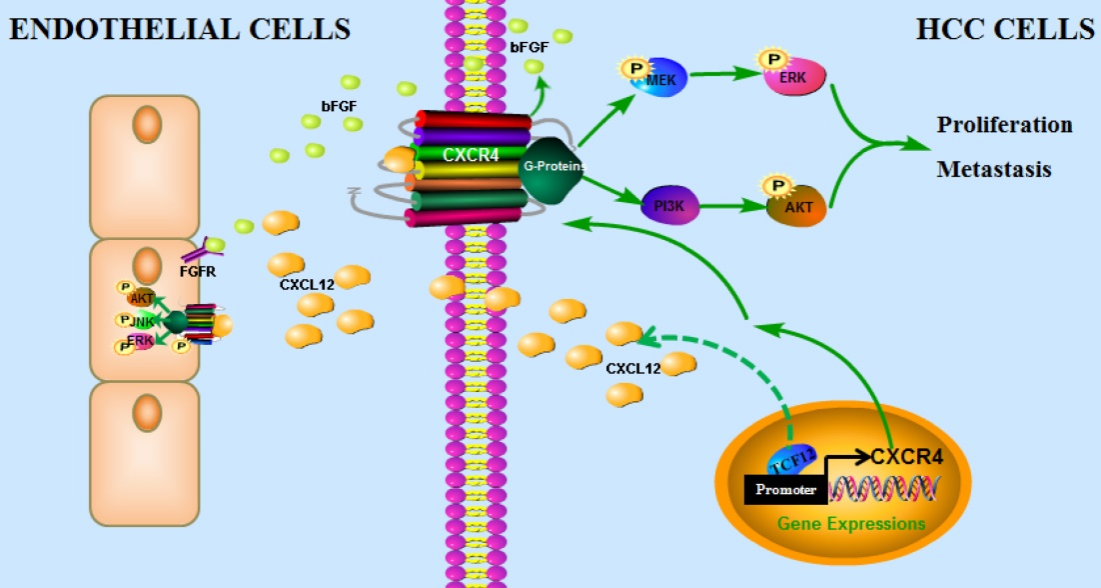
** The proposed model illustrates how the transcription factor TCF12 promotes MAPK/ERK and PI3K/AKT signaling in HCC.** TCF12 exerts its oncogenic activity by upregulating CXCR4 expression, thereby activating MAPK/ERK and PI3K/AKT signaling in HCC cells. In addition, TCF12 participates in the angiogenesis of endothelial cells in the tumor microenvironment via regulation of CXCL12 and CXCR4-mediated bFGF expression.

In conclusion, our findings have uncovered a novel mechanism by which the CXCL12-CXCR4 axis is involved in pro-tumor function of TCF12 in HCC, leading to the activation of the MAPK and PI3K-AKT signaling pathways and thereby promoting HCC cell growth, migration and invasion, which provides a novel target and a valuable prognostic marker for HCC. In addition, TCF12 participates in the angiogenesis of endothelial cells in the tumor microenvironment via regulation of CXCL12 and CXCR4-mediated bFGF expression (Figure 9).

## Supplementary Material

Supplementary figures and tables.

## Abbreviations

AKT: protein kinase B
bHLH: basic helix-loop- helix
CXCR4: CXC chemokine receptor 4
CXCL12: C‑X‑C chemokine ligand 12
ERK: extracellular signal- regulated kinase
EMT: epithelial-mesenchymal transition
HCC: hepatocellular carcinoma
IHC: immunology and histology chemistry
MTT: 3-(4,5-dimethylthiazol-2-yl)-2,5-diphenyltetrazolium bromide
NC: negative control
RT-qPCR: quantitative real-time polymerase chain reaction
SDS-PAGE: sodium dodecyl sulphatepolyacrylamide gel electrophoresis
shRNA: short hairpin RNA
TCF12: transcription factor 12
TCGA: the cancer genome atlas
TSS: transcriptional start site
