# Supplementary material for: TCF12 promotes the tumorigenesis and metastasis of hepatocellular carcinoma via upregulation of CXCR4 expression
Source: Theranostics. 2019 Aug 12;9(20):5810–27. doi: 10.7150/thno.34973 (PMC6735379; doi:10.7150/thno.34973)
Supplement: Supplementary file 1 — Supplementary figures and tables. [file thnov09p5810s1.pdf]

Supplementary figures (Figure S1-S8)

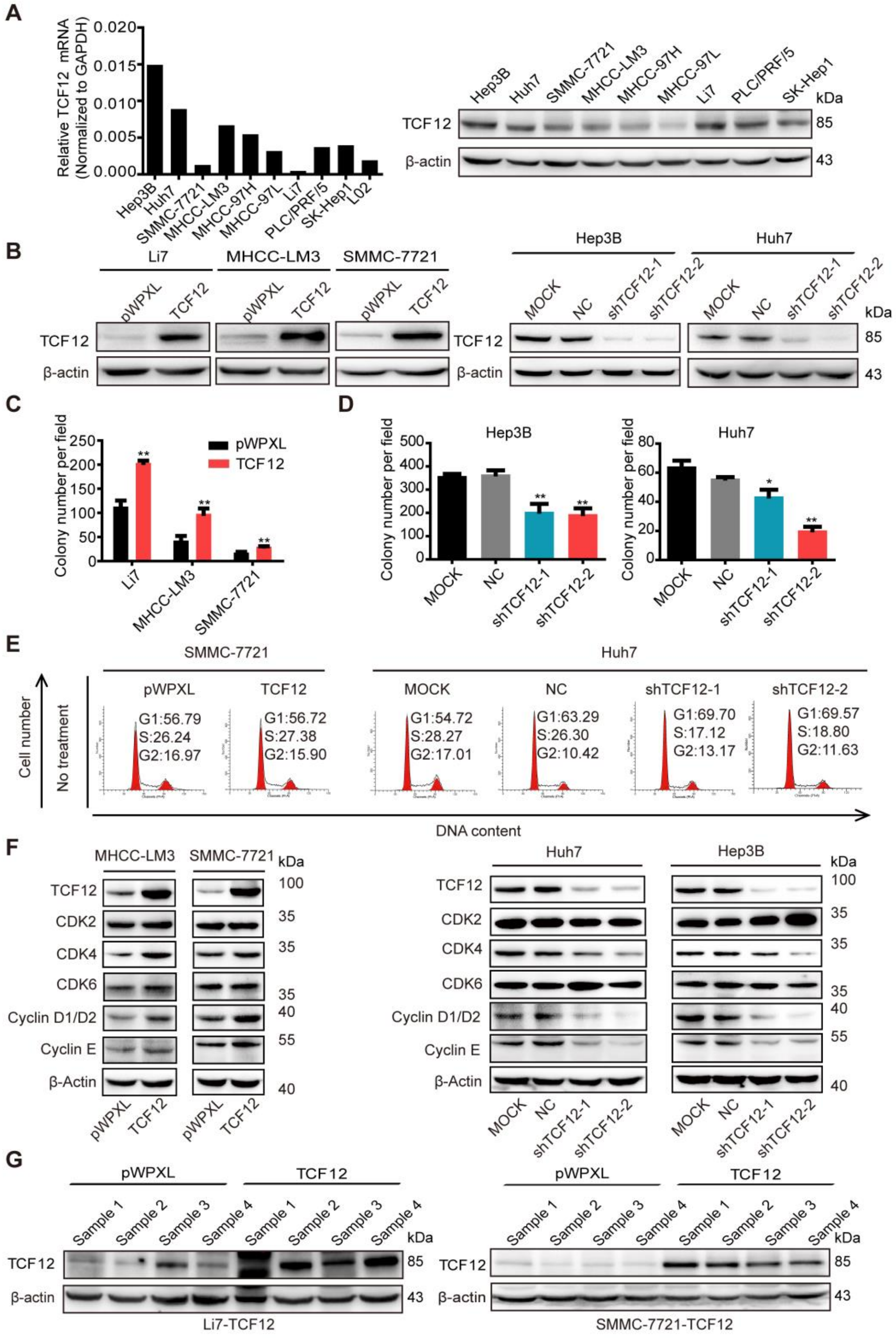

Figure S1. TCF12 promotes HCC cell proliferation. (A) RT-qPCR (left panel) and western blot analysis (right panel) of TCF12 expression in HCC cells and the immortalized normal hepatocyte cell line L02. **(B)** Overexpression of TCF12 in Li7,

MHCC-LM3 and SMMC-7721 cells and knockdown of TCF12 in Hep3B and Huh7 cells were confirmed by western blot. pWPXL, empty vector**;** MOCK, blank control; NC: negative control. **(C, D)** The summary graphs were presented for the colony formation of HCC cells with TCF12 overexpression or knockdown. The bar graphs represent quantitative data from three replicates. **(E)** Cell cycle profile for TCF12 overexpression in SMMC-7721 and TCF12 knockdown in Huh7 cells were analyzed by FACS. **(F)** Western blot analysis of cell cycle related proteins in TCF12-overexpressed and knockdown cells. **(G)** The protein level of TCF12 in xenograft tissues with Li7 and SMMC-7721 cells stably transfected with empty vector pWPXL or TCF12 were detected by western blot. β-actin was used as a loading control. Data are shown as the mean ±S.D. from experiments with three replicates. *$P < 0.05$, **$P < 0.01$.

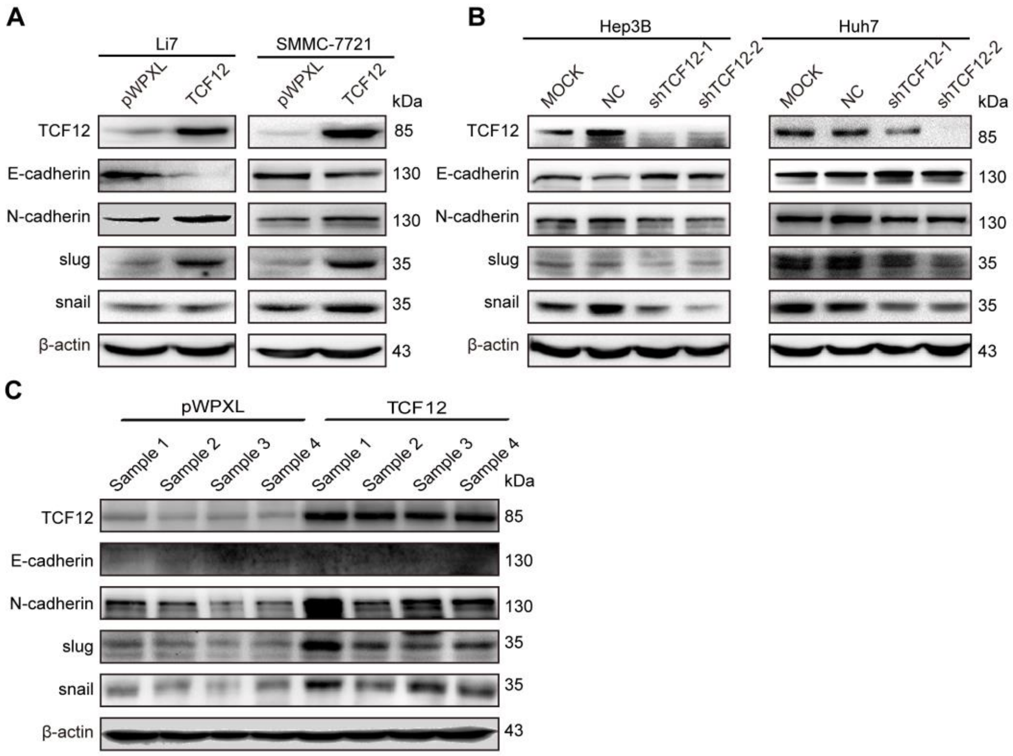

Figure S2. TCF12 induces EMT in HCC cells. (A) Western blot analysis of EMT markers in TCF12 overexpressing Li7 and SMMC-7721 cells, and the corresponding controls. **(B)** Western blot analysis of EMT markers in TCF12 knockdown Hep3B and Huh7 cells, and the corresponding controls. **(C)** Western blot analysis of EMT

markers in xenograft tissues formed by SMCC-7721 cells.

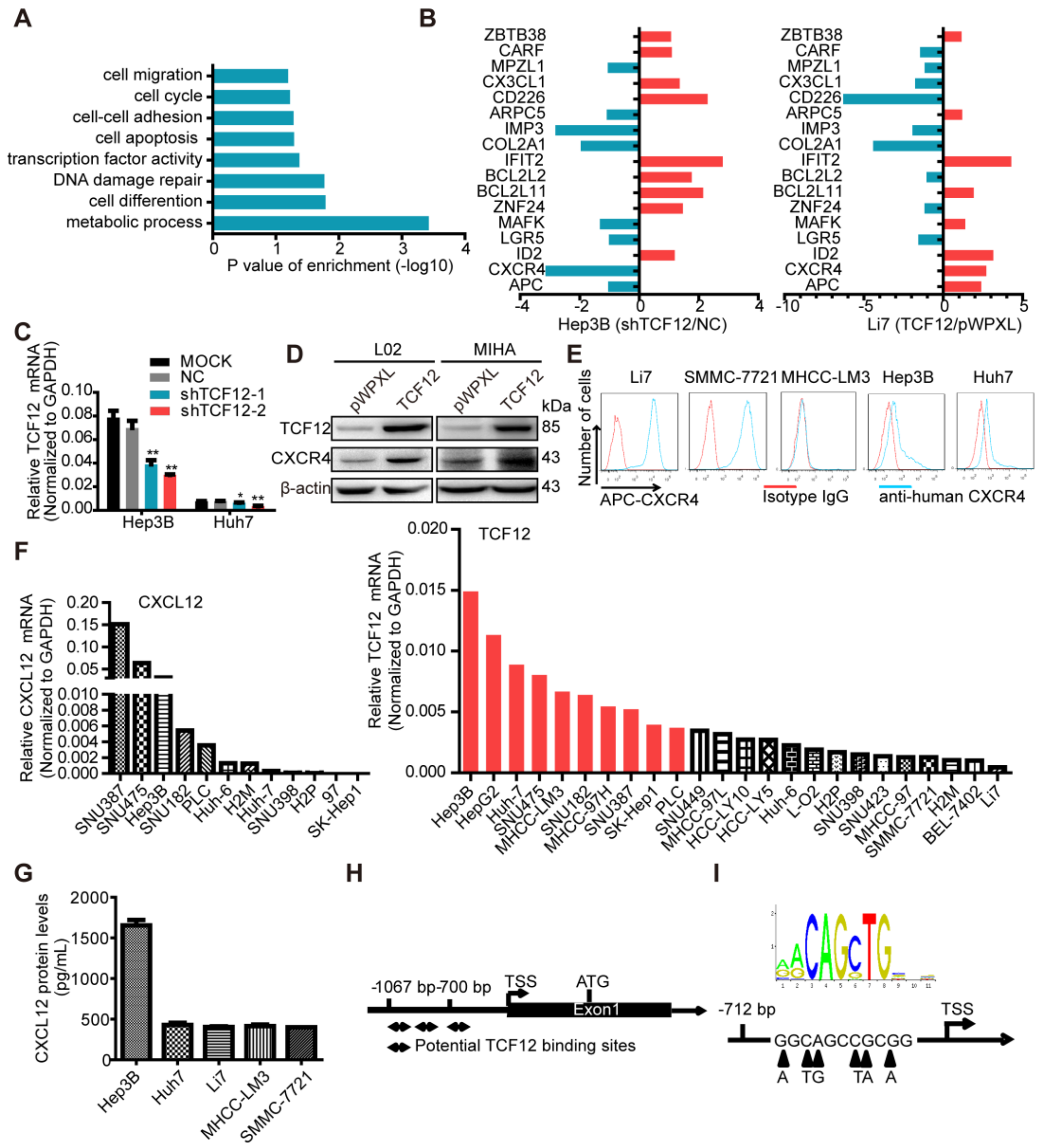

Figure S3. TCF12 regulates CXCR4 and CXCL12 expression. (A) Gene ontology analyses of TCF12-regulated gene expression events. Fisher exact *P* values were plotted for each category. **(B)** The mRNA expression levels of multiple selected HCC-related genes among the differentially expressed genes from the RNA-Seq results were evaluated by RT-qPCR. **(C)** The detection of mRNA levels of TCF12 in Hep3B and Huh7 cells after TCF12 knockdown as a control for Figure 3B. **(D)** The protein levels of TCF12 and CXCR4 in L02 and MIHA cells expressing pWPXL empty vector or TCF12 were detected by western blot. **(E)** The percentage of

$CXCR4^+$ cells among the HCC cell lines was analyzed by flow cytometry analysis. **(F)** RT-qPCR was used to detect TCF12 and CXCL12 mRNA expression in 24 HCC cells. The red bars in the right panel represent the HCC cell lines with a relatively high mRNA expression of TCF12. **(G)** The CXCL12 protein level in the culture medium was measured by ELISA in the five HCC cells used in our study. The bar graph represents quantitative data from three independent experiments. **(H)** Schematic diagram illustrating the TCF12-targeted sites on the CXCR4 promoter as predicted on the JASPAR website. **(I)** The sequence of the TCF12 potential binding site on the CXCR4 promoter in JASPAR (upper panel) and a diagram of the mutated predicted binding site (lower panel). Data are shown as the mean ± S.D. from experiments with three replicates. $*P < 0.05$, $**P < 0.01$.

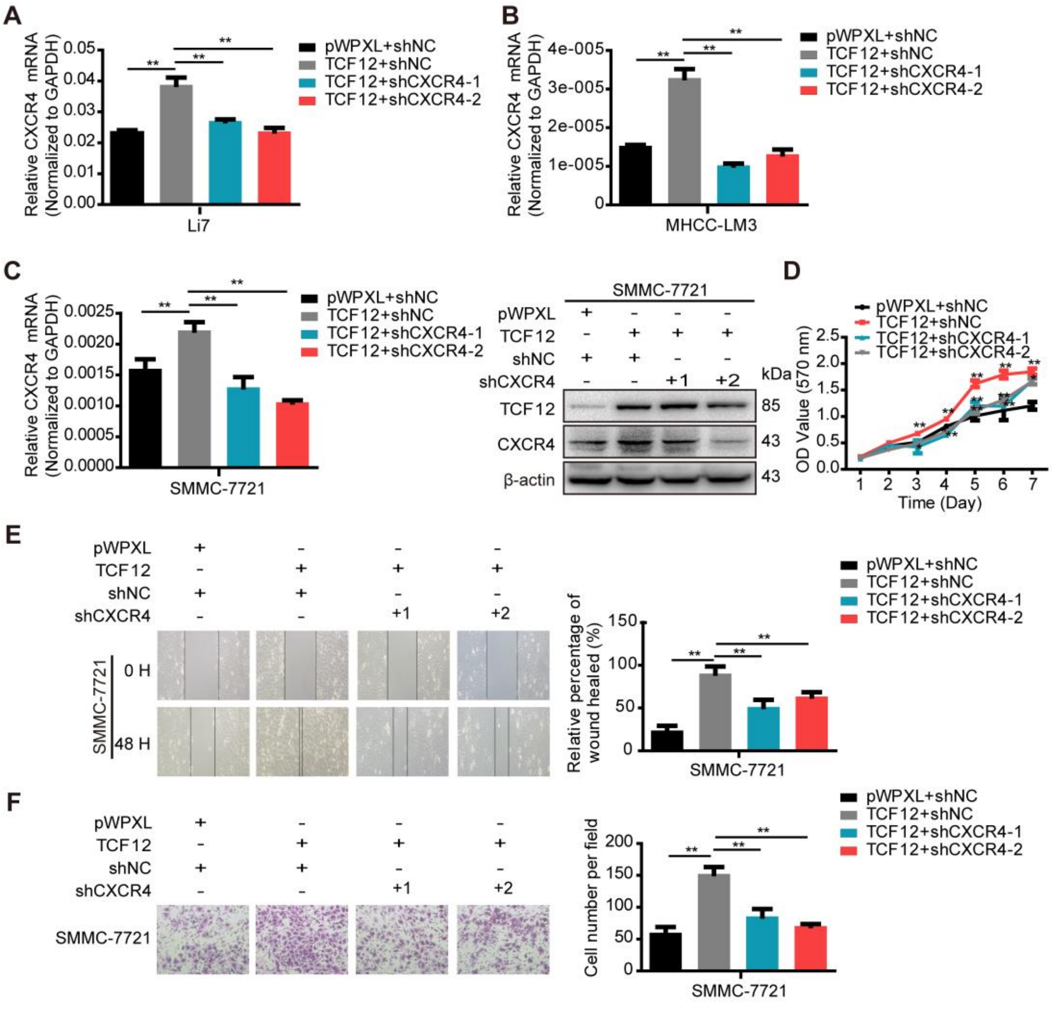

Figure S4. CXCR4 silencing inhibits the pro-oncogenic capacity induced by

TCF12 overexpression in HCC cells. (A, B) Li7 and MHCC-LM3 cells that stably expressed TCF12 were transfected with either shNC or shRNA targeting CXCR4, and the mRNA knockdown efficiency of CXCR4 was detected by RT-qPCR. **(C)** CXCR4 knockdown reversed the upregulation of CXCR4 mRNA (left panel) and protein levels (right panel) induced by TCF12 overexpression in SMMC-7721 cells. **(D)** The influence of silencing CXCR4 on the effects of TCF12-induced growth in MIHA cells was measured by the MTT assay. **(E, F)** Representative micrographs of the influence of CXCR4 knockdown on the effects of TCF12-induced migration and invasion in SMMC-7721 cells. Data are shown as the mean ± S.D. from experiments with three replicates. $^{**}P < 0.01$.

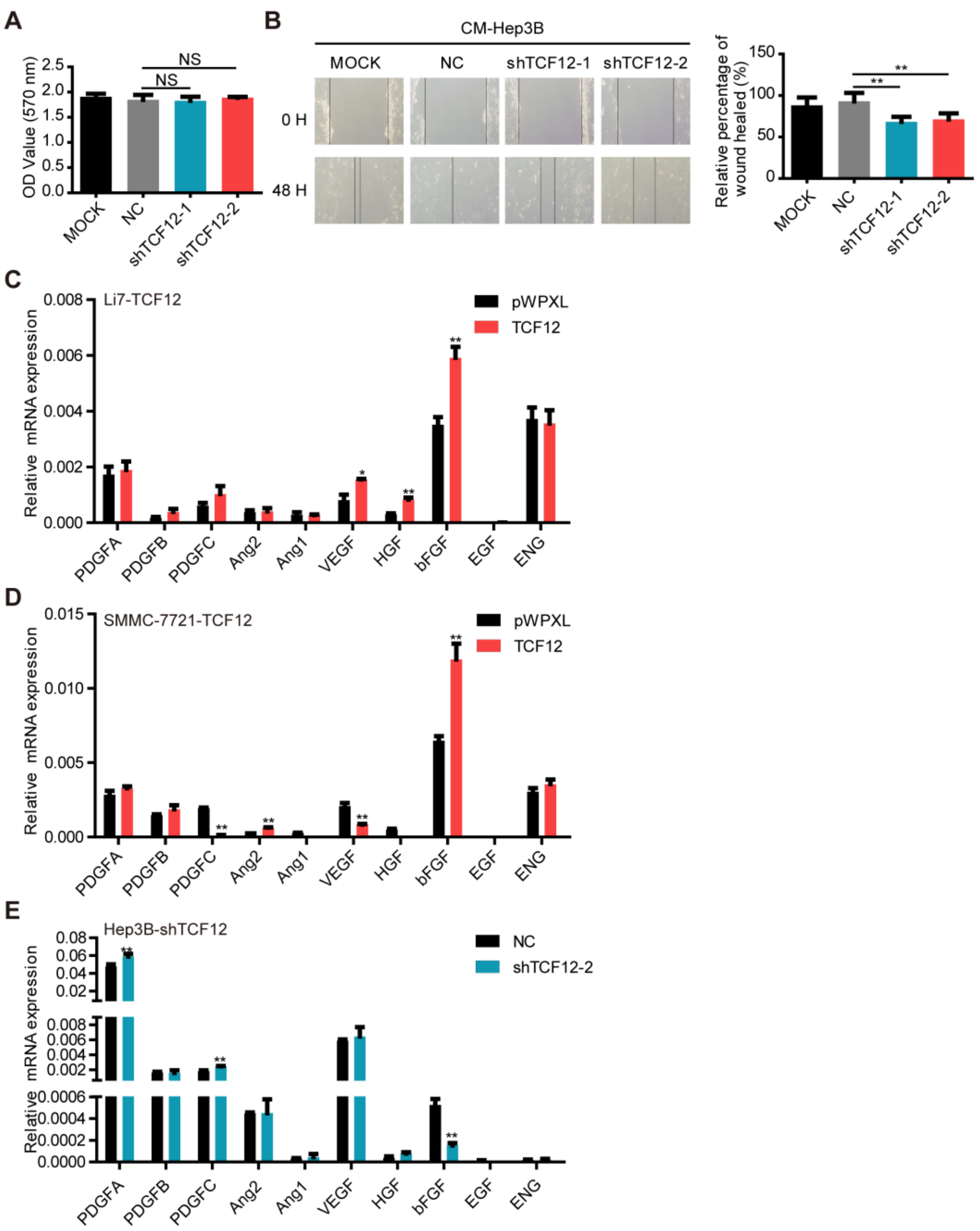

Figure S5. TCF12 promotes tumor angiogenesis. (A) MTT assay of HUVECs after treatment for 48 h with CM from Hep3B-MOCK, Hep3B-shNC or Hep3B-shTCF12. **(B)** Representative micrographs of the *in vitro* wound healing assay of HUVECs after treatment with CM from the above mentioned Hep3B cells. The bar graph (right panel) shows quantitative analysis data with three replicates. **(C-E)** RT-qPCR was used to detect 10 kinds of common angiogenic factors in TCF12-overexpressed Li7 and SMMC-7721 cells and TCF12-silenced Hep3B cells.

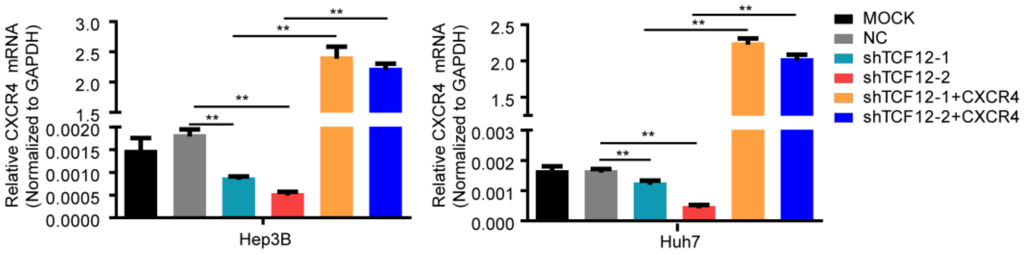

Figure S6. Overexpression of CXCR4 in Hep3B and Huh7 cells with TCF12 knockdown was confirmed by RT-qPCR.

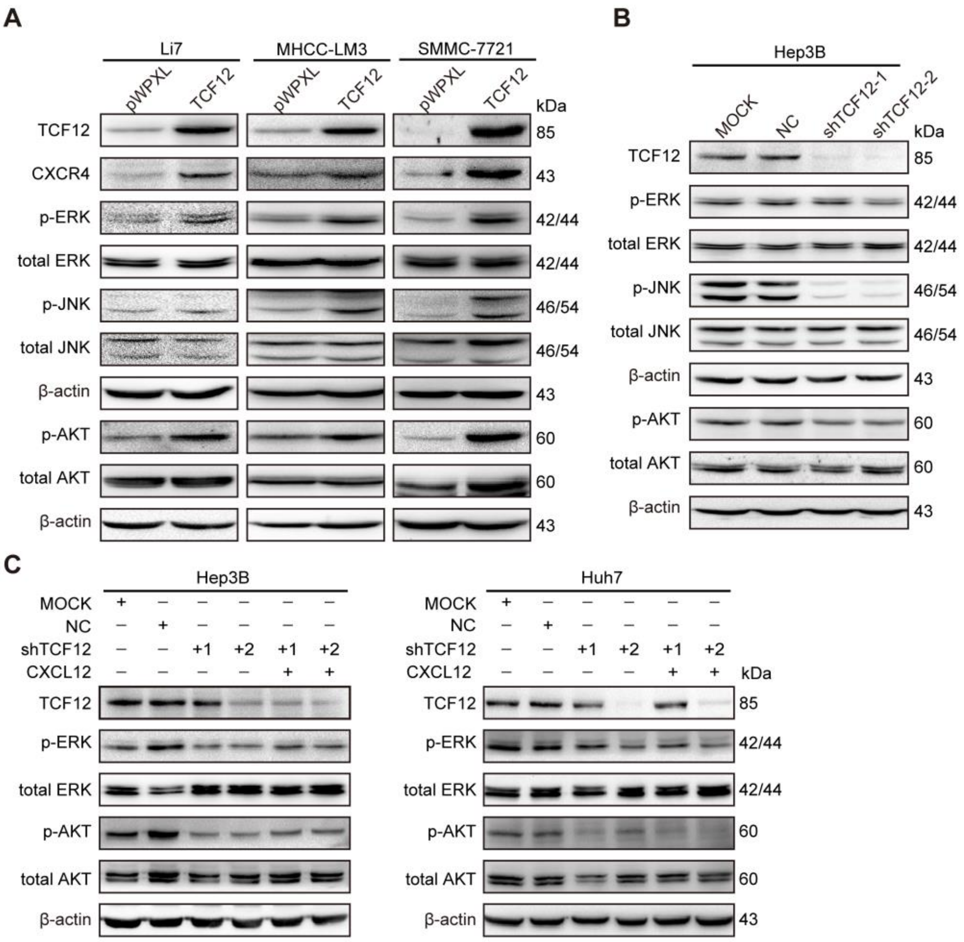

Figure S7. TCF12 can activate downstream pathways of MAPK and PI3K/AKT signaling. (A) Western blot analysis of the levels of phosphorylated ERK, JNK and AKT in Li7, MHCC-LM3 and SMMC-7721 cells transfected with either empty vector pWPXL or TCF12. **(B)** Western blot analysis of the levels of phosphorylated ERK, JNK and AKT in Hep3B cells transfected with shNC or shTCF12. **(C)** Knockdown of TCF12 reduced phosphorylation of MAPK/ERK and PI3K/AKT pathway proteins, but phosphorylation levels were restored weakly in Hep3B cells or remained unchanged

in Huh7 cells exposed to CXCL12 protein (100 ng/mL). β-actin were used as loading controls.

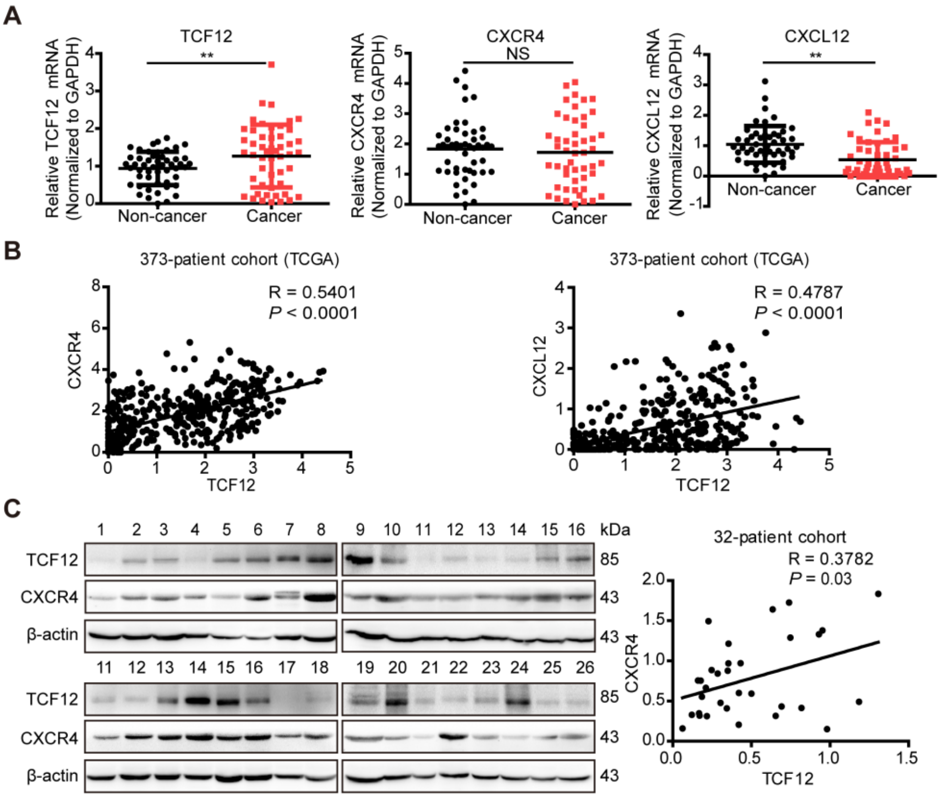

Figure S8. TCF12 mRNA expression is correlated with CXCR4 and CXCL12 mRNA expression in HCC tissues. (A) TCF12, CXCR4 and CXCL12 mRNA expression in 50 paired HCC tissues and noncancerous liver tissues in the TCGA cohort (left panel, TCF12; middle panel, CXCR4; right panel, CXCL12). **(B)** Scatter plots showing the correlation between TCF12 mRNA levels and CXCR4 or CXCL12 mRNA levels in 373 HCC samples from the TCGA cohort (R, Pearson correlation coefficient; *P*, p value). **(C)** Western blot analysis of the TCF12 and CXCR4 protein levels in 26 HCC tissues. Scatter plots in the right panel showing the correlation between TCF12 and CXCR4. ***P* < 0.01, NS: no significance.

Supplementary tables (Table S1-S8)

Table S1. The primer and shRNA sequence used for this study

**Name**	**Primer Sequence (5'-3')**
TCF12-F	AGGTGGCTTGCAAAGTCAGT
TCF12-R	AGTACTGCTTGTTCTGCCTCT
GAPDH-F	AGAAGGCTGGGGCTCATTTG
GAPDH-R	AGGGGCCATCCACAGTCTTC
CXCR4-F	CTTCTTAACTGGCATTGTGG
CXCR4-R	GTGATGACAAAGAGGAGGTC
CXCL12-F	TCAGCCTGAGCTACAGATGC
CXCL12-R	CTTTAGCTTCGGGTCAATGC
APC-F	GACTCGGAAATGGGGTCCAA
APC-R	TCTTCAGTGCCTCAACTTGCT
ID2-F	TCCCACTATTGTCAGCCTGC
ID2-R	AGAAGCCTGCAAGGACAG
LGR5-F	AACATCAGTCAGCTGCTCCC
LGR5-R	CATCCAGACGCAGGGATTGA
MAFK-F	CGACTAATCCCAAACCGAAT
MAFK-R	ACATGGACACCAGCTCATCA
ZNF24-F	ATCTTGGAGCTGGTAGTGCTGGAG
ZNF24-R	GCACTGTCACTGCCTCCTCTCC
BCL2L11-F	AAGAGTTGCGGCGTATTGGAGAC
BCL2L11-R	ACCAGGCGGACAATGTAACGTAAC
BCL2L2-F	GCGGAGTTCACAGCTCTATACGG
BCL2L2-R	CAGCACTGTCCTCACTGATGCC
IFIT2-F	GCGTGAAGAAGGTGAAGAGGAAGG
IFIT2-R	TGGCTGCACTGCGAAGAACATC
COL2A1-F	GGAGCAGCAAGAGCAAGGAGAAG
COL2A1-R	TGGACAGCAGGCGTAGGAAGG
IMP3-F	GGACTACACGCGCTACAACCAG
IMP3-R	GCACCAAGCCGAGAGCATACAG
TIMP2-F	ACGAGTGCCTCTGGATGGACTG
TIMP2-R	GGAGCCGTCACTTCTCTTGATGC
ARPC5-F	GAGTCAGGCAGTGAAGGACC
ARPC5-R	ACTGCTATTGTCAGACGGGC
CD226-F	GGCAGAAGGTGATACAGGTGGTTC
CD226-R	CTGAGGCTGACAAGTGAGTGTGAC

CX3CL1-F	GAAGCAGATCGGCGAGGTGAAG
CX3CL1-R	GGAGTCGGCTCCAGGCTACTG
MPZL1-F	TTGACCTCAGTCTCCTGGAGCTTC
MPZL1-R	TGTCCGCCGGAGTGGTCTAAC
CARF-F	CCAGCCGTCCTCTAGTCCTTCAG
CARF-R	AACTTCTACCAGCTCGTCCATTGC
ZBTB38-F	AGCCAACCCAGGAGCCTTTA
ZBTB38-R	AGGAATGGGCATGTGGCTTG
Ang2-F	AACTTTCGGAAGAGCATGGAC
Ang2-R	CGAGTCATCGTATTCGAGCGG
Ang1-F	AGCGCCGAAGTCCAGAAAAC
Ang1-R	TACTCTCACGACAGTTGCCAT
HGF-F	GCTATCGGGGTAAAGACCTACA
HGF-R	CGTAGCGTACCTCTGGATTGC
bFGF-F	AGAAGAGCGACCCTCACATCA
bFGF-R	CGGTTAGCACACACTCCTTTG
EGF-F	TGTCCACGCAATGTGTCTGAA
EGF-R	CATTATCGGGTGAGGAACAACC
ENG-F	GCATCCTTCGTGGAGCTACC
ENG-R	GAGGAGTGGTCTGGATCGG
PDGFA-F	CCCCTGCCCATTCGGAGGAAGAGA
PDGFA-R	TTGGCCACCTTGACGCTGCGGTG
PDGFB-F	CTCGATCCGCTCCTTTGATGA
PDGFB-R	CGTTGGTGCGGTCTATGAG
PDGFC-F	GACTCAGGCGGAATCCAACC
PDGFC-R	CTTGGGCTGTGAATACTTCCATT
TCF12-Clone-F	ATGAATCCCCAGCAACAACG
TCF12-Clone-R	TTACATATGACCCATAGGGTTGG
shNC	TTCTCCGAACGTGTCACGT
shTCF12-1	ATCCCATAATGCACCAATT
shTCF12-2	TGTGATTATGGTGAACATA
CXCR4-Clone-F	ATGTCCATTCCTTTGCCTCT
CXCR4-Clone-R	TTAGCTGGAGTGAAAACTTG
shCXCR4-1	AAGGAACCCTGTTTCCGTGAA
shCXCR4-2	CTCCTTCATCCTCCTGGAAAT
CXCR4 (-1067)-F	GTCCTGCAGTTCGAGAGTTTG
CXCR4 (-712)-F	TGTGGGACAGAGCCTGGCG
CXCR4-Mut-F	TGTGGGACAGAGCCTGGCGTGTCGCCCAGCGGAGC

	CCCTGCAGCGCTGCTTGCGGGCGGTTGGCGTGGGT
	GTAGTGAGTGGCTACAGCGGC
CXCR4 (universal)-R	CTGAAGTAGTGGGCTAAGGGC
CXCR4-CH-IP-F	CGGTTGGCGTGGGTGTAGT
CXCR4-CH-IP-R	ACTGATCCAGTTAACCCGGCC

Table S2. The antibodies for western blot used in this study

Antibody	Source	Catalogue number	Dilution	Application	Company
**Primary antibodies for WB**					
TCF12	Mouse IgG	sc28364	1:250	WB	Santa Cruz
CXCR4	Rabbit IgG	ab124824	1:250	WB	abcam
CDK2	Rabbit IgG	SC-163	1:200	WB	Santa Cruz
CDK4	Mouse IgG	SC-23896	1:200	WB	Santa Cruz
CDK6	Mouse IgG	SC-7961	1:200	WB	Santa Cruz
CyclinD1/D2	Goat IgG	AF4196	1:200	WB	R&D
CyclinE	Rabbit IgG	SC-481	1:200	WB	Santa Cruz
E-cadherin	Rabbit IgG	sc7870	1:250	WB	Santa Cruz
N-cadherin	Rabbit IgG	22018-1-AP	1:1000	WB	proteintech
Slug	Rabbit IgG	CST9585	1:250	WB	Cell signaling
Snail	Mouse IgG	CST3895	1:250	WB	Cell signaling
bFGF	Rabbit IgG	CST3196	1:250	WB	Cell signaling
p-ERK1/2	Rabbit IgG	CST9101	1:1000	WB	Cell signaling
ERK1/2	Rabbit IgG	CST4695	1:1000	WB	Cell signaling
p-JNK	Rabbit IgG	CST4668	1:1000	WB	Cell signaling
JNK2	Rabbit IgG	CST9258	1:1000	WB	Cell signaling
p-AKT	Rabbit IgG	CST3787	1:1000	WB	Cell signaling
AKT	Rabbit IgG	CST4691	1:1000	WB	Cell signaling
**Secondary antibodies for WB**					
HRP-anti-Rabbit IgG	Goat	A0545	1:3000	WB	Sigma-Aldrich
HRP-anti-Mouse IgG	Goat	A4416	1:5000	WB	Sigma-Aldrich

Table S3. The antibodies for the IHC used in this study

Antibodies	Source	Catalogue number	Dilution	Application	Company
**Primary antibodies for IHC**					
TCF12	Rabbit IgG	ab91592	1:25	IHC	Abcam
CXCR4	Rabbit IgG	ab227767	1:10	IHC	Abcam
CD34	Mouse IgG	550537	1:50	IHC	BD Biosciences
**Secondary antibody for IHC**					
HRP-anti-Rat IgG	Rabbit	A5795	1:50	IHC	Sigma-Aldrich
EnViSion (Peroxidase/DAB; Rabbit/Mouse)	Detection	Kit K5007		IHC	DAKO

Table S4.The cell cycle distribution of SMMC-7721 and Huh7 cells after TCF12 overexpression or knockdown

			SMMC-7721(%)		Huh7(%)			
			PWPXL	TCF12	MOCK	NC	shTCF12-1	shTCF12-2
No treatment		G1	56.66±0.30	56.75±0.74	53.49±1.25	62.58±2.23	71.16±2.25**	71.09±2.24**
		S	27.63±2.37	28.84±4.34	29.64±2.98	25.36±0.84	15.61±2.59**	17.13±1.44**
		G2/M	15.7±2.07	14.41±3.64	16.86±3.38	12.06±2.76	13.23±0.38	11.78±1.78
Synchronization with thymidine	0h	G1	63.10±1.62	64.58±2.69	57.23±2.64	58.48±0.89	64.17±4.98	61.09±4.15
		S	30.30±2.96	28.12±3.05	32.59±4.91	30.93±2.68	28.16±3.49	32.42±4.48
		G2/M	6.60±1.51	7.31±0.36	10.18±2.35	10.59±1.90	7.67±1.59	6.49±0.37
	12h	G1	72.07±0.31	72.04±1.24	27.67±2.42	26.36±1.11	49.62±2.97**	56.06±6.72**
		S	7.89±1.76	8.33±0.99	15.77±4.75	20.57±6.75	24.16±1.81	11.09±7.23
		G2/M	20.04±2.04	19.63±0.27	56.55±7.17	53.07±7.84	26.21±2.91**	32.86±1.21*
	24h	G1	72.49±0.70	68.44±0.79**	64.53±2.45	70.97±1.98	66.57±2.46	79.16±1.31**
		S	19.30±0.31	23.78±0.77**	22.67±2.04	21.58±1.28	17.79±1.00*	13.2±2.32**
		G2/M	8.21±0.58	7.78±0.96	12.80±4.40	7.45±3.27	15.64±2.44*	7.64±1.06

Data are shown as the mean ± S.D. from experiments with three replicates.

$*P < 0.05$, $**P < 0.01$, comparing to the control pWPXL or NC group.

Table S5. Summary of liver metastasis and lung metastasis in mice

Nudemice numbered	Number of liver metastasis		Number of lung metastasis	
	pWPXL	TCF12	pWPXL	TCF12
1	3	9	0	2
2	4	3	0	0
3	2	9	0	1
4	6	5	0	3
5	4	8	0	4
6	4	12	0	0

Table S6. Selected differentially expressed genes after TCF12 silenced in Hep3B cells based on RNA-Seq

Functional gene groups	Symbol	Descrption	Fold Change
**Cell proliferation**	APC	adenomatous polyposis coli	5.35
	CXCR4	Chemokine (C-X-C motif) receptor 4	-3.74
	ID2	inhibitor of DNA binding 2	-2.22
	LGR5	leucine-rich repeat containing G protein-coupled receptor 5	-2.18
	MAFK	v-maf avian musculoaponeurotic fibrosarcoma oncogene homolog K	-4.14
	ZNF24	zinc finger protein 24	4.39
**Cell apoptosis**	BCL2L11	BCL2-like 11	3.45
	BCL2L2	BCL2-like 2	-2.70
	IFIT2	interferon induced protein with tetratricopeptide repeats 2	4.58
**Cell migration**	COL2A1	collagen, type II, alpha 1	-3.46
	CXCR4	Chemokine (C-X-C motif) receptor 4	-3.74
	IMP3	U3 small nucleolar ribonucleoprotein	-4.92
	TIMP2	TIMP metallopeptidase inhibitor 2	2.54
**Cell adhesion**	ARPC5	actin related protein 2/3 complex subunit 5	-2.28
	CD226	CD226 molecule	6.55
	CX3CL1	chemokine (C-X3-C motif) ligand 1	-2.74
	MPZL1	myelin protein zero-like 1	2.55
**DNA damage repair**	CARF	CDKN2A interacting protein	-2.08
	ZBTB38	zinc finger and BTB domain containing 38	-2.03

Table S7. Relationship between TCF12 protein expression and clinicopathological parameters in HCC patients

Clinicopathological Features	Number of cases	TCF12 expression		*P* Value
		Low N (%)	High N (%)	
Age (years)				
< 50	129	89 (66.42)	40 (71.43)	0.500
≥ 50	61	45 (33.58)	16 (28.57)	
HBsAg				
Negative	32	22 (17.05)	10 (17.86)	0.894
Positive	153	107 (82.95)	46 (82.14)	
Anti-HBs				
Negative	168	119 (95.97)	49 (90.74)	0.164
Positive	10	5 (4.03)	5 (9.26)	
HBeAg				
Negative	144	103 (83.06)	41 (77.36)	0.372
Positive	33	21 (16.94)	12 (22.64)	
Anti-HBe				
Negative	96	71 (57.72)	25 (45.45)	0.129
Positive	82	52 (42.28)	30 (54.55)	
Anti-HBc				
Negative	33	21 (17.07)	12 (22.22)	0.418
Positive	144	102 (82.93)	42 (77.78)	
AFP (ng/mL)				
< 20	65	41 (31.30)	24 (42.11)	0.152
≥ 20	123	90 (68.70)	33 (57.89)	
Tumor size				
< 5cm	99	69 (53.08)	30 (53.57)	0.951
≥ 5cm	87	61 (46.92)	26 (46.43)	
Histological grade				
I, II	94	60 (44.78)	34 (59.65)	0.06
III, IV	97	74 (55.22)	23 (40.35)	
Cirrhosis				
Absent	33	24 (17.91)	9 (15.79)	0.723
Present	158	110 (82.09)	48 (84.21)	
Intrahepatic metastasis				
Absent	138	95 (70.90)	43 (75.44)	0.521
Present	53	39 (29.10)	14 (24.56)	

AFP: alpha-fetoprotein; N: Number of cases; *P* value represents the probability from Chi-square test for different immunohistochemical scores of TCF12 in HCC tissues.

Table S8. Relationship between CXCR4 protein expression and clinicopathological parameters in HCC patients

Clinicopathological Features	Number of cases	TCF12 expression		*P* Value
		Low N (%)	High N (%)	
Age (years)				
< 50	129	98 (70.00)	31 (62.00)	0.298
≥ 50	61	42 (30.00	19 (38.00)	
HBsAg				
Negative	32	23 (16.91)	9 (18.37)	0.817
Positive	153	113 (83.09)	40 (81.63)	
Anti-HBs				
Negative	168	125 (94.70)	43 (93.48)	0.757
Positive	10	7 (5.30)	3 (6.52)	
HBeAg				
Negative	144	109 (82.58)	35 (77.78)	0.475
Positive	33	23 (17.42)	10 (22.22)	
Anti-HBe				
Negative	96	75 (56.82)	21 (45.65)	0.191
Positive	82	57 (43.18)	25 (54.35)	
Anti-HBc				
Negative	33	23 (17.42)	10 (22.22)	0.475
Positive	144	109 (82.58)	35 (77.78)	
AFP (ng/mL)				
< 20	65	43 (31.16)	22 (44.00)	0.102
≥ 20	123	95 (68.84)	28 (56.00)	
Tumor size				
< 5cm	99	74 (54.01)	25 (51.02)	0.718
≥ 5cm	87	63 (45.99)	24 (48.98)	
Histological grade				
I, II	94	63 (44.68)	31 (62.00)	0.035*
III, IV	97	78 (55.32)	19 (38.00)	
Cirrhosis				
Absent	33	29 (20.57)	4 (8.00)	0.043*
Present	158	112 (79.43)	46 (92.00)	
Intrahepatic metastasis				
Absent	138	98 (69.50)	40 (80.00)	0.154
Present	53	43 (30.50)	10 (20.00)	

AFP: alpha-fetoprotein; N: Number of cases; *P* value represents the probability from Chi-square test for different immunohistochemical scores of CXCR4 in HCC tissues. * $P < 0.05$.
